# Controlled Release Utilizing Initiated Chemical Vapor Deposited (iCVD) of Polymeric Nanolayers

**DOI:** 10.3389/fbioe.2021.632753

**Published:** 2021-01-28

**Authors:** Karen K. Gleason

**Affiliations:** Department of Chemical Engineering, Massachusetts Institute of Technology, Cambridge, MA, United States

**Keywords:** drug delivery systems, initiated chemical vapor deposition, thin films, nanofibers, conformal coating, smart materials, thermally responsive polymers, pH responsive polymers

## Abstract

This review will focus on the controlled release of pharmaceuticals and other organic molecules utilizing polymeric nanolayers grown by initiated chemical vapor deposited (iCVD). The iCVD layers are able conform to the geometry of the underlying substrate, facilitating release from one- and two-dimensional nanostructures with high surface area. The reactors for iCVD film growth can be customized for specific substrate geometries and scaled to large overall dimensions. The absence of surface tension in vapor deposition processes allows the synthesis of pinhole-free layers, even for iCVD layers <10 nm thick. Such ultrathin layers also provide rapid transport of the drug across the polymeric layer. The mild conditions of the iCVD process avoid damage to the drug which is being encapsulated. Smart release is enabled by iCVD hydrogels which are responsive to pH, temperature, or light. Biodegradable iCVD layers have also be demonstrated for drug release.

## Introduction

Controlling the rate and location of drug release in the body allows for the customization of therapeutic regimens for improved efficacy and outcomes (Peppas et al., 2006; Lau and Gleason, 2007; McInnes et al., 2012; Coclite, 2013; Sayin et al., 2019a). For some chronic treatments, slow-release can be desired over a long period of time. More acute conditions may require larger doses delivered quickly to a targeted location at one or more points in time. Limiting release rates can minimize toxic side effects. Using polymers to encapsulate drugs has been widely utilized to achieve controlled release. In some cases, the polymer layer erodes or dissolves away, introducing a time delay before the encapsulated drug is released at a rate comparable to the uncoated drug (Shi et al., 2018). In other situations, the polymer layer remains and presents a permeation barrier to slow the diffusion of therapeutic molecules from the encapsulated drug reservoir (Coclite, 2013; Gleason, 2020a). In the case where the polymer layer remains, the drug molecules contained in the encapsulated reservoir must diffuse through the polymer layer before being released in the body. The rate of release will depend on the thickness of the polymer layer, with thinner layers allowing for a higher release rate. Faster release rates also result from increasing the total surface area available for drug release. The total surface area can be increased by utilizing non-planar interfaces. Additionally, the release rate will be determined by the molecular architecture within the polymeric encapsulation layer.

While liquid-phase methods are commonly used to encapsulate drugs for controlled release applications, vapor phase methods are emerging (Perrotta et al., 2018; Yu et al., 2018). One vapor-phase strategy that provides ultrathin encapsulation of drug reservoirs from complex geometries is initiated Chemical Vapor Deposition (iCVD) polymerization. By using a low-growth temperature and by avoiding the use of solvent, the iCVD method enables polymers to form directly on fragile substrates without degrading them. Indeed, pharmaceuticals have been demonstrated to retain their efficacy after being encapsulated with iCVD layers (Sayin et al., 2019b; Decandia et al., 2020).

The iCVD process brings nanoscale control to polymer thin film synthesis. Using controlled iCVD growth rates allows the formation of pinhole-free films as thin as 10 nm (Gleason, 2020a,b). Alternatively, utilizing processing conditions that result in high deposition rates allows iCVD film thicknesses of 1,000 nm or more to be deposited in a reasonable period (Shi et al., 2018). Ultrathin (<20 nm) and ultrasmooth (<1 nm rms roughness) CVD polymers can conform to the geometry of the growth surface (Moni et al., 2017). The conformality of iCVD polymers enables the full encapsulation of medical devices that can have complex geometries. Demonstrations of controlled release from non-planar geometries include the drug release from textiles employed in wound dressings (Ghasemi-Mobarakeh et al., 2019), drug crystallites (Lau and Gleason, 2007), porous membranes (Shi et al., 2018), and polymeric nanostructures (Ozaydin-Ince et al., 2011).

The iCVD method couples the versatility of organic chemistry with the high-purity and systematic process control of all-dry vacuum processing (Gleason, 2015; Wang et al., 2017a; Matsumura et al., 2019). Surfaces in direct contact with living cells and tissues must be free of impurities and stable under the conditions of use (Donadt and Yang, 2020). A vapor-deposition approach to surface modification eliminates the possibility of contamination by any residual solvent. Additionally, the process of purifying iCVD monomers, which are small molecules, is much easier than purifying macromolecular polymer chains.

## iCVD Polymer Composition and Design

Some monomers utilized to date for the iCVD synthesis of controlled release layers are shown in Table 1. The list of monomers in Table 1 is a subset of the >70 monomers that have been polymerized by iCVD so far (Gleason, 2020b). The organic functional groups present in the monomers are fully retained in the corresponding iCVD polymer. The functional groups are responsible for the properties of the polymeric layers. In particular, a high level of functional group retention is critical for achieving smart, responsive behavior. Numerous iCVD polymers and copolymers have been integrated into a variety of geometries for controlled release (Table 2). Post-deposition modification of the iCVD functional groups provides additional options to achieve precise chemical signaling at the surface. The resulting iCVD film compositions represent hundreds of different homopolymers, copolymers, and crosslinked organic networks (Gleason, 2020b). Rather than competing with commonly used solution methods, iCVD offers the ability to extend the available operating window available for the applications of polymeric thin films.

**Table 1:** Selected iCVD monomers.

**iCVD monomer name**	**Acryonym**	**Functional group**	**Example film characteristics**	**References**
**MONOVINYL**				
4-vinyl-pyridine	4VP	Pyridine	pH-responsive hydrogels	Ghasemi-Mobarakeh et al., 2019; Sayin et al., 2019b
1H,1H,2H,2H-perfluorodecyl acrylate	PFDA	Perfluoro	Hydrophobic barrier	Christian et al., 2016
2-hydroxyethylmethacrylate	HEMA	Hydroxyl	Hydrophilic, hydrogel former	Yang et al., 2012; Tufani and Ince, 2015
2-hydroxypropyl methacrylate	HPMA	Hydroxyl	Hydrophilic, hydrogel former	Sevgili and Karaman, 2019
cyclohexyl methacylate	CHMA	Cyclohexyl	Hydrophobic barrier	Bose et al., 2012a
Ethyl acrylate	EA	Ethyl	Used as component of an enteric copolymer	Lau and Gleason, 2007
Glycidyl methacrylate	GMA	Epoxy	Hydrophobic barrier, surface functionalization	Bose et al., 2012a
Methacrylic acid	MAA	Carboxylic acid	Surface reactive, pH responsive	Lau and Gleason, 2007; McInnes et al., 2012; Ghasemi-Mobarakeh et al., 2019
N-isopropylacrylamide	NIPAAm	Secondary amide	Thermally responsive hydrogels	Armagan and Ozaydin Ince, 2015; McInnes et al., 2016; Werzer et al., 2019
N-vinylcaprolactam	NVCL	Caprolactam	Thermally responsive hydrogels	Yin et al., 2012
N,N-dimethyacrylamide	DMAAm	Tertiary amide	Thermally responsive hydrogels	Tenhaeff and Gleason, 2009
Tert-butyl acrylate	tBA	Tert-butyl	Shape-memory polymers	Ozaydin-Ince et al., 2011
**MULTIVINYL (CROSSLINKERS)**				
di(ethylene glycol) divinyl ether	DEGDVE	Crosslinking, ether	Mechanically robust	Tenhaeff and Gleason, 2009; McInnes et al., 2016; Werzer et al., 2019
Divinylbenzene	DVB	Crosslinking, hydrophobic	Mechanically robust	Oh et al., 2016
Ethylene glycol diacrylate	EGDA	Crosslinking, acrylate	Mechanically robust	Lau and Gleason, 2007; Ozaydin Ince et al., 2010
Ethylene glycol dimethacrylate	EGDMA	Crosslinking, methacrylate	Mechanically robust	Christian et al., 2016, 2018; Gleason, 2020a; Mansurnezhad et al., 2020
Methacrylic anhydride	MAH	Crosslinking, anhydride	pH sensitive, biodegradable	Tenhaeff and Gleason, 2009; Shi et al., 2018; Decandia et al., 2020
2,4,6-trivinyl-2,4,6-trimethyl cyclotrisiloxane	V3D3	Crosslinking, cyclic siloxane	Low k dielectric, biocompatable	O'Schaughnessy et al., 2007

**Table 2:** Selected examples of iCVD layers for controlled release.

**Polymer composition**	**As-deposited thickness (nm)**	**Substrate**	**Permeant**	**References**
**NEUTRAL HYDROGELS**				
P(HEMA-co-EGDA)	40	Porous alumina membranes	Fluorescein (dye)	Ozaydin-Ince et al., 2011
P(HEMA-co-EGDMA)	30–60	Porous alumina membranes	Phloroglucinol (dye)	Armagan and Ozaydin Ince, 2015
P(HEMA-co-EGDMA)	200	Solid drug layer on a planar substrate	Indomethacin (pain relief)	Christian et al., 2018
P(HEMA-co-EGDMA)	200–500	PET, cotton fabric, polycaprolactone nanofiber mats	Indomethacin (pain relief)	Ghasemi-Mobarakeh et al., 2019
P(HEMA-co-EGDMA)	200	solid drug layer on a planar substrate	Clotimazole (antifungal)	Christian et al., 2016
P(HEMA-co-EGDMA)	1,000	Free-standing films released from polyacrylic acid sacrificial layer	Alizarin yellow; phloroglucinol; 4-phenyldiphenylamin (dyes)	Tufani and Ince, 2015
**pH RESPONSIVE**				
P(MAA-co-EA)	70–400	ibuprofen microcrystals	ibuprofen (pain relief)	Lau and Gleason, 2007
P(MAA-co-EDMA)	340	Porous silicon loaded with drug	Camptothecin (cancer treatment)	McInnes et al., 2012
P(MAA-co-EGDMA)	200	Solid drug layer on a planar substrate, cellulose membrane loaded with drug	Gentamicin (antibiotic)	Decandia et al., 2020
P(MAA-co-EGDMA)	200	Solid drug layer on a planar substrate	Clotimazole (antifungal)	Christian et al., 2016
P(MAA-co-EGDMA)	200–500	Drug loaded PET, cotton fabric, polycaprolactone nanofiber mats	Indomethacin (pain relief)	Ghasemi-Mobarakeh et al., 2019
P(MAA-co-DMAAm-co-DEGDVE)	70–500	AAO membranes	Glucose (sugar); bovine serum albumin (protein)	Tenhaeff and Gleason, 2009
P(MAH-co-MAA) (conformal)	1,000	Drug-loaded microporous polylactide membranes	Rifampicin (antibiotic)	Shi et al., 2018
P(4VP-co-EGDMA)	65	Mat of polyvinyl alcohol nanofibers electrospun with RB	Rose Bengal (RB) (chemotherapeutic agent)	Sayin et al., 2019b
**THERMALLY RESPONSIVE**				
P(NIPAAm-co-EGDMA)	30–60	AAO template	phloroglucinol (dye)	Armagan and Ozaydin Ince, 2015
P(NIPAAm-co-DEGDVE)	200	Porous silicon loaded with drug	Camptothecin (cancer treatment)	McInnes et al., 2016
P(NIPAAm-co-DEGDVE)	200	Solid drug layer on a planar substrate	Clotimazole, indomethacin (pain relief) phenytoin	Werzer et al., 2019
P(NVCL-co-DEGDVE)	10–50	Planar Eudragit layer	na	Muralter et al., 2019
**HYDROPHOBIC ENCAPSULATION**				
P(CHMA-co-EDGMA)	400–600	Solid microparticles of an agrochemical	Crop protection compound	Bose et al., 2012a
PEGDMA	200	Solid drug layer on a planar substrate	Indomethacin (pain relief)	Christian et al., 2018
PEGDMA	23	Mat of gelatin nanofibers	Gelatin	Mansurnezhad et al., 2020
PPFDA	200	Solid drug layer on a planar substrate	Clotimazole (antifungal)	Christian et al., 2016

The crosslinked hydrogel networks formed by iCVD are of high interest for controlled drug release (Lau and Gleason, 2007; Ghasemi-Mobarakeh et al., 2019). The diverse library of compositions allows iCVD polymers (Table 2) to match the properties required for mass transport through the semipermeable hydrogel layers (Gleason, 2020a). The mesh architecture of the hydrogel controls determine the ease of permeability for different molecular species of varied size, shape, and polarity through the iCVD film. The expanded mesh structures of crosslinked iCVD hydrogel polymer layers swollen in aqueous media allows for the ready passage of permeant molecules, including drugs and agrochemicals. Kinetic studies of the controlled release process sometimes use model compounds. Dyes are particularly valuable as model compounds since the release of the dye into solution is easy to monitor by optical measurements.

The iCVD synthesis method provides precise mechanistic control over polymer chain formation, crosslinking, and morphology, all of which are all critical for controlling average mesh size. Systematic variation in iCVD process parameters provides remarkable control over molecular architecture. Characteristics length scales can be tuned with sub-0.1 nm precisions for the average mesh sizes of crosslinked iCVD hydrogels (Table 3) (Yagüe and Gleason, 2012; Armagan and Ozaydin Ince, 2015; Unger et al., 2016). The mesh openings allow the permeation of the species to be released (Gleason, 2020a). Larger mesh dimensions increase the release rate. Increasing mesh size can result from decreasing the crosslinker fraction and increasing the length of the bridging moiety in crosslinker molecule. The average mesh size can be calculated from the degree of swelling using the Flory-Rehner theory (Peppas et al., 2006).

**Table 3:** Average mesh size calculated from the measured swelling of iCVD hydrogels.

**Mono-vinyl monomer(s)**	**Crosslinking monomer**	**Geometry**	**% crosslinker**	**Mesh size (nm)**	**References**
**NON-HYDROGELS**					
na	EGDMA	Film on Si	100	0.1	Christian et al., 2018
**NEUTRAL HYDROGELS**					
HEMA	EGDMA	Film on Si	58	0.15	Christian et al., 2018
HEMA	EGDMA	Film on Si	55	0.18	Christian et al., 2018
HEMA	EGDMA	Film on Si	27	0.29	Christian et al., 2018
HEMA	EGDMA	Free-standing	32	0.57	Tufani and Ince, 2015
HEMA	EGDMA	Free-standing	47	0.65	Tufani and Ince, 2015
HEMA	EGDA	Film on Si	69	0.45	Yagüe and Gleason, 2012
HEMA	EGDA	Film on Si	67	1.5	Yagüe and Gleason, 2012
HEMA	EGDA	Film on Si	57	2.0	Yagüe and Gleason, 2012
HEMA	DEGDVE	Film on Si	na	3.1	Mao et al., 2018
**pH RESPONSIVE HYDROGELS**					
MAA at pH 4	EGDMA	Film on Si	56	0.83	Armagan and Ozaydin Ince, 2015
MAA at pH 8	EGDMA	Film on Si	56	3.2	Armagan and Ozaydin Ince, 2015
4VP at pH 9	EGDMA	Film on Si	na^*^	0.69	Sayin et al., 2019b
4VP at pH 6.5	EGDMA	Film on Si	na^*^	0.69	Sayin et al., 2019b
4VP at pH 4	EGDMA	Film on Si	na^*^	1.2	Sayin et al., 2019b
MA (14%) and dimethyl acrylamide (76%) at pH 7	DEGDVE	Film on Si	10	3.2	Tenhaeff and Gleason, 2009
**THERMORESPONSIVE HYDROGELS**					
NIPAAm at 25°C	DEGDVE	Film on Si	7.5 ± 2.5	6.0	Alf et al., 2010, 2011
NIPAAm at 25°C	EGDMA	Film on Si	23	1.4	Armagan and Ozaydin Ince, 2015
NIPAAm at 40°C	EGDMA	Film on Si	23	0.71	Armagan and Ozaydin Ince, 2015

* *same value but difficult to calculate*.

## iCVD Polymer Properties

The mechanistically-based iCVD approach provides full retention of a monomer's organic functional groups and thus enables a rational basis for designing and optimizing film characteristics. The full retention of organic functional groups by iCVD polymerization is essential for the fabrication of smart layers capable of switching permeation behavior in response to variations in light, pH, or temperature (Gleason, 2020a,b). The ability to synthesis biodegradable and biocompatible iCVD polymers is also desired for controlled release applications requiring systems that can be implanted into the body.

**Figure 1:**
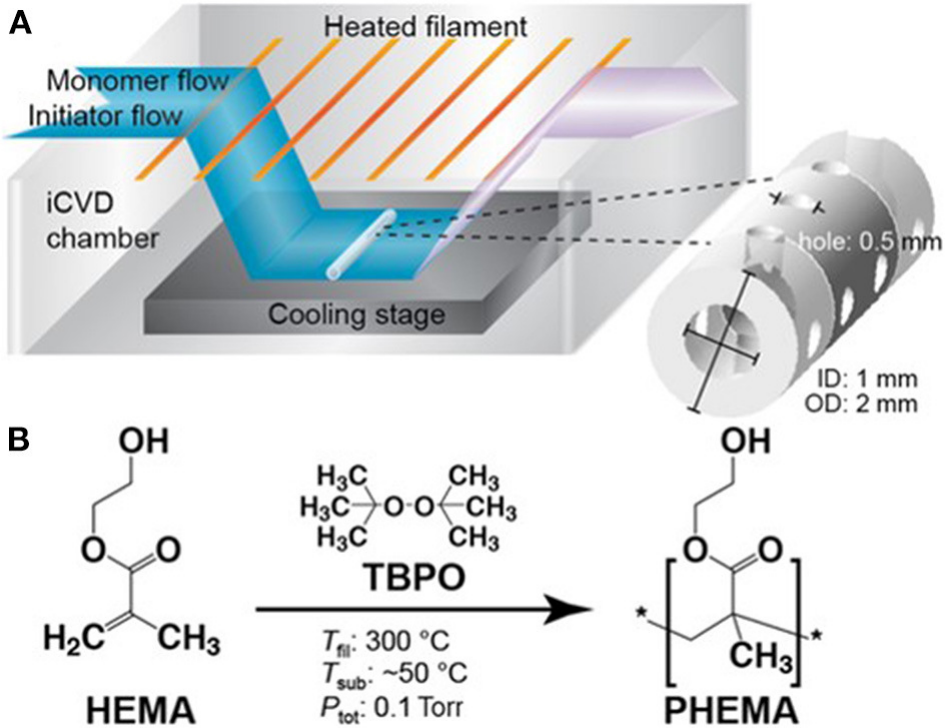
**(A)** Schematic of an iCVD chamber operating at modest vacuum. The reactants enter the chamber as vapors. The initiator thermal decomposes at or near the heated filaments and the monomer adsorbs and polymerizes on the cooled stage. Selecting process conditions for achieving conformal iCVD growth allows all the surface of geometrically complex substrates to be uniformily covered. **(B)** Chemical structures of the vinyl monomer HEMA, the initiator TBPO, and the homopolymer formed by iCVD along with typical temperatures for the filament, substrate, and chamber pressure. Reprinted with permission Hanak et al. (2018).

The degree of swelling by neutral hydrogels typically shows limited response external pH and temperature values. Neutral hydrogel layers synthesized from the monomer HEMA (Figure 1) are biocompatibility and resistant biofouling (Peppas et al., 2006). Their swelling behavior is driving by hydrophilic hydroxyl (-OH) group of the HEMA monomeric unit. The growth and properties of iCVD of HEMA-based hydrogels have been extensively studied (Yang et al., 2012; Mao et al., 2018). Other neutral hydrogels, such as those based on the monomer hydroxypropyl methacrylate, have also been successfully synthesized by iCVD (Sevgili and Karaman, 2019).

The incorporation methacrylic acid (MAA) monomer units into a hydrogel imparts pH responsive swelling and, thus, pH-responsive controlled release behavior (Lau and Gleason, 2007; Ghasemi-Mobarakeh et al., 2019). The carboxylic acid functional (-COOH) group present in MAA is sensitive to changes in pH. At low pH, the –COOH remain protonated and thus MAA-containing polymers are charge neutral and display limited swelling in water. At high pH, the carboxylic acid group deprotonates to –COO^−^, thus switching the MAA-containing polymers to their anionic form which swells more easily in water.

Since MAA-based hydrogel layers display enhanced swelling under basic conditions, these materials are useful for enteric release. Enteric coatings are dense polymers at low pH, protecting orally administered drugs from the acid environment of the stomach. Once the drug passes through to the higher pH environment of the lower gastrointestinal tract, the enteric coating swells or degrades, allowing the drug to be released. Commercially available enteric coatings include Eudragit (Rohm), Kollicoat (BASF), and Eastacryl (Eastman Chemical) (Lau and Gleason, 2007). These products are copolymers containing MAA units and are applied by spray-coating.

Hydrogel layers containing 4-vinyl-pyridine (4VP) are also pH-responsive but their behavior is opposite to that of an enteric coating. The 4VP-based hydrogel layers display enhanced swelling in acidic environment, because the pyridine functional group converts to its conjugate acid form at low pH (Ghasemi-Mobarakeh et al., 2019; Sayin et al., 2019b). The cationic nature of 4VP-containing polymers at low pH enhances their swelling. At higher pH, the 4VP reverts to its neutral form and exhibits limited swelling in water.

Thermally responsive hydrogel encapsulation for controlled drug release has been achieved utilizing the N-isopropylacrylamide (NIPAAm) monomer (Werzer et al., 2019). The barrier to drug diffusion presented by NIPAAm-containing films switches with temperature. Above their lower critical solution temperature (LCST), the film is dense and rate of drug release is slow. Lowering the temperature below the LCST, causes the film to swell with water (Alf et al., 2010, 2011). This swelling facilitates the diffusion of the drug across the iCVD layer, thus enhancing the release rate. The transition at the LCST results because hydrogen bonding between the polymer and water is thermodynamically favored at low temperature, while hydrogen bonding between different polymer chains becomes favored at high temperature and water is expelled. Polymers containing NIPAAm have LCST values near body temperature, making them of particular interest for medical applications.

One strategy for drug release uses coating of a drug a stable permselective polymer For this approach, the release rate is controlled by the diffusion of the drug across the thickness of the polymer layer. Using iCVD, polymer layers can be produced at thicknesses ranging from <10 nm to >100 μm (Gleason, 2020a). Additionally, the iCVD method provides the ability to grow layers free of pinholes. Pinhole defects are undesirable because they can allow rapid and uncontrolled release of the underlying drug.

An alternative strategy for controlled drug release is using an polymeric encapsulation layer which erodes away in a controllable manner. After the initial delay required for complete erosion, the drug will be rapidly released as a result of being directly exposed to the aqueous solution. Erosion of an iCVD layer to achieve controlled release has been demonstrated results by utilizing methacrylate anhydride (MAH) as an iCVD monomer (Shi et al., 2018). The MAH moieties incorporated into the film are subject to hydrolysis, resulting in conversion to MAA units along the backbone. As the concentration of MAA units in the polymer increase, the iCVD layer becomes increasingly water soluble, causing the film to erode.

The commercial introduction of any new material or synthesis method requires regulatory approval to ensure human safety and to protect the environment (Perrotta et al., 2018). Solution applied polymer coatings often contain small molecule impurities in the form of residual solvent, unreacted monomer, degradation products, and additives designed to improve coating quality and uniformity. Often these impurities, rather than the macromolecular chains, that are responsive for failures in biocompatibility testing (Bhat, 2002). Since no solvent or additives are used, CVD polymers there are few types of potential impurities, only unreacted initiator or monomer and degradation products. Multiple iCVD polymers have been demonstrated to be cytocompatible. No cytotoxic effects were seen in rat embryo fibroblasts cultured on functionalized HEMA-based iCVD hydrogels (Unger et al., 2017), or in PC12 neurons cultured on pV3D3 (O'Schaughnessy et al., 2007). The stability of the pV3D3 under 3 years of soaking at physiological conditions was also reported (O'Schaughnessy et al., 2007). Long-term culture and survival of primary hippocampal neurons was demonstrated for iCVD poly(2-(dimethyamino)ethyl methacrylate) (Yu et al., 2016). For iCVD poly(glycidal methacrylate), no cytotoxicity was observed toward adipose derived stem cells and the stability of this iCVD coating on titanium was confirmed by implantation into pigs (Park et al., 2015). These studies demonstrate initial promise, but much more extensive study is needed.

## iCVD Polymerization

For iCVD polymerization, vapors of one or more monomers and an initiator species polymerize on a surface. The iCVD process synthesizes a macromolecular film in a single step (Gleason, 2015; Wang et al., 2017a). Most commonly, thermally activated free-radical initiators are used for iCVD polymerization. Film growth by iCVD polymerization has also be demonstrated by cationic initiation (Bose et al., 2012b; Gao et al., 2018), photoinitiation (Chan and Gleason, 2005; Martin et al., 2007), or low-power plasma initiation (Boscher et al., 2016; Loyer et al., 2018).

### Reaction Chamber and Process Conditions

A schematic of a typical lab-scale iCVD reaction chamber is displayed in Figure 1 (Hanak et al., 2018). The chamber is held at moderate vacuum levels, typically between 0.1 and 1.0 torr. Vapors of one or more monomer species and vapors of the initiator mix inside the chamber. An array of resistively heated filament wires inside the chamber causes the thermal decomposition of the initiator molecules to form free radical species. The relative modest filament temperatures, typically between 200 and 350°C, produces little, if any, decomposition of the monomers.

The growth stage of the iCVD chamber is cooled to maintain a low substrate temperature, typically in the range ~25–65°C. Cool substrates promote the adsorption of the monomers, as is required for heterogeneous polymerization. The low substrate temperature of iCVD is also compatible with substrates that are unable to withstand significant heating. As a result, iCVD layers have been deposited directly on top of reservoir layers of drug molecules without degrading the drug (Decandia et al., 2020).

Chain growth polymerization proceeds via a heterogeneous surface reaction between the impinging volatile initiator radicals with adsorbed monomers (Mao and Gleason, 2004; Matsumura et al., 2019) The iCVD monomers shown in Table 1 polymerize through their vinyl bonds (>C=C<). For monomers having only one vinyl bond, such as 2-hydroxyethyl methacrylate (HEMA) linear polymer chains result (Figure 1). The polymeration of monomers with more than one vinyl bond will be discussed in the next section. For most iCVD monomers, the vinyl polymerization produces an all-*sp*^3^ carbon backbones that do not biodegrade. One exception is the polymerization of MAH, which results in the incorporation of a biodegradable anhydride group into the backbone of the iCVD polymer chain.

An essential parameter for controlling the iCVD process is the monomer saturation ratio (SR) (Lau and Gleason, 2006a,b). The SR is the dimensionless ratio of the partial pressure of the monomer to its saturation pressure at the growth temperature. Liquid condensation occurs at SR = 1. The isotherms describing monomer adsorption depend directly on SR. The film growth rate, number average molecular weight of the polymer chains, of the iCVD film all strongly influenced SR. Lower the SR values increase the conformality of coverage by iCVD films.

In many cases, iCVD utilizes SR values between 0.3 and 0.5, the same range anticipated for monolayer adsorption of the monomer (Lau and Gleason, 2006a,b). Since vapor pressure is a widely measured and estimated property, the understanding of the importance of SR has allowed the rapid expansion of iCVD synthesis for more than 70 monomers (Gleason, 2019).

**Figure 2:**
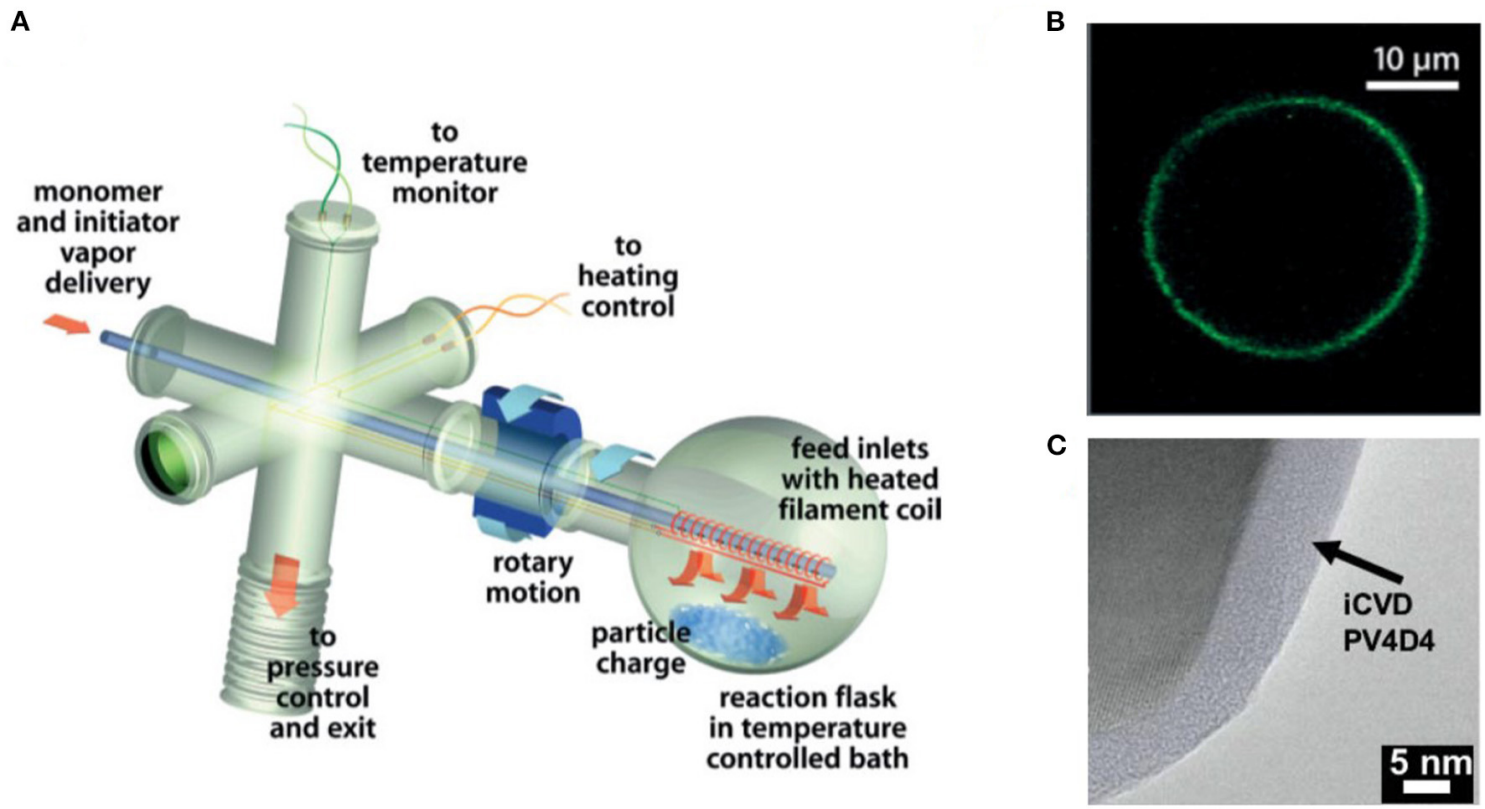
**(A)** Schematic of rotating bed iCVD reactor created by customizing a rotary evaporator. **(B)** Micrograph of an conformal polymer coating on glass microbead grown by iCVD in the rotating bed reactor and then functionized with a fluorescent dye. **(C)** Transmission electron micrograph (TEM) of an ultrathin, ultrasmooth iCVD crosslinked polymer conformally grown around a solid particle. Parts **(A)** and **(B)** reprinted with permission from Lau and Gleason (2006c) and part **(C)** reprinted with permission from Moni et al. (2017).

Motivated by lower the cost per unit area, iCVD deposition has been demonstrated for the fabrication of large-area polymer thin films and operation by roll-to-roll processing (Gupta and Gleason, 2006; Pryce Lewis et al., 2009; Kovacik et al., 2015; Cheng and Gupta, 2018; Yilmaz et al., 2019). Additionally, innovation is reducing the cost of laboratory-scale CVD polymer reactors (Randall et al., 2018). The design of the iCVD reactor determines the geometry of the substrates that can be coated. For example, a rotating bed reactor was designed to coat batches of solid powders (Figure 2) (Lau and Gleason, 2006c). With the optimized iCVD process parameters, including SR, ultrathin iCVD polymers conformally deposit on microparticles (Moni et al., 2017).

### Copolymerization and Crosslinking

Many of iCVD films demonstrated for controlled drug release applications are copolymers (Table 2). Instead of feeding a single type of monomer vapor, which produces a homopolymer, simultaneously feeding two or more types of monomers into the iCVD chamber forms copolymers. Controlling the ratio of the types of monomers in the vapor feed produces systematic compositional changes in the resulting iCVD copolymers. Because monomers differ in their ease of adsorption to the growth surface and in their reactivity with like and unlike types of monomers, the ratio of monomers in the vapor feed typically differs the ratio present in the iCVD film that forms. The dependence of iCVD copolymer composition on the selected vapor feed ratio can be quantitatively analyzed using the Fineman-Ross equation (Lau and Gleason, 2007).

Incorporating monomers with two or more vinyl bonds produces crosslinked iCVD films. In solution polymerization, macromolecule growth, film formation, and crosslinking often occur as three distinct steps. In contrast, the iCVD method produces crosslinked films from their monomers in a single processing step. A crosslinking monomer can be homopolymerized to produce a covalent organic network. For example, Table 2 contains an example of a crosslinked homopolymer grown from the divinyl monomer, ethylene glycol dimethacrylate (EGDMA).

Crosslinking typically enhances the durability of the iCVD films. For example, homopolymerization 2,4,6-trivinyl-2,4,6-trimethylcyclotrisiloxane (V3D3) results in highly crosslinked iCVD network. The iCVD PV3D3 displays remarkable stability, providing unchanged biopassivation properties while immersed in physiological saline at 37°C for several years of observation (O'Schaughnessy et al., 2007). Thus, the PV3D3 films are attractive as stable coatings on biomedical implants. Crosslinking also often decreases rms roughness of iCVD films to sub-nanometer values (Gleason, 2020a).

Table 2 reveals the iCVD crosslinked film more commonly are grown by copolymerizing a multivinyl monomer with a monovinyl monomer. For iCVD employing hydrophilic monovinyl monomers, the corresponding linear homopolymer chains can partially or fully dissolve when placed in an aqueous environment. Forming copolymers of the hydrophilic monomers with crosslinkers can inhibit dissolution of the iCVD film in aqueous media.

Typically, a limited degree of crosslinking is desired for controlled release applications. Reducing the crosslinking fraction increases the average mesh size of the crosslinked network (Table 3). Larger mesh sizes increase the diffusion of drug molecules through the iCVD layer. Using a low fraction of crosslinker also allows the properties of the copolymer film to most closely align with the characteristics of the homopolymer grown from the monovinyl monomer. However, the crosslinking fraction must remain sufficient to inhibit the film from dissolving in aqueous solutions.

**Figure 3:**
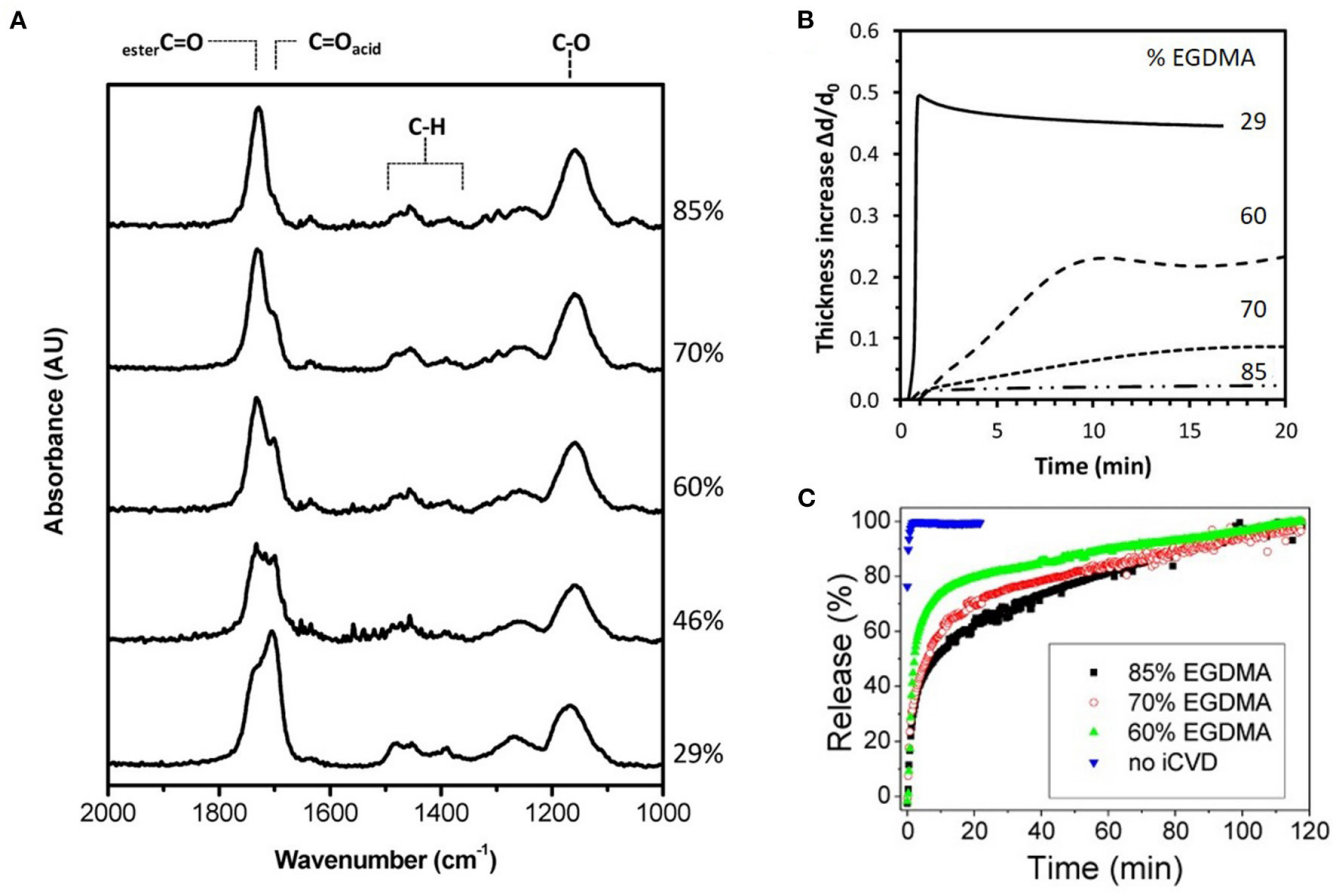
Composition, swelling dynamics, and release kinetics for the iCVD copolymer P(MAA-co-EGDMA) with labels showing the vol.% of the EGDMA crosslinker in the film. **(A)** The Fourier Transform Infrared (FTIR) spectra with the carbonyl peak at ~1,700 cm^−1^ having resolvable contributions from both monomers. **(B)** The dynamics of swelling shown as the thickness increase due to swelling in water normalized by the initial thickness of the dry iCVD film as a function of time. **(C)** Percentage of gentamicin released with time. Reprinted with permission from Decandia et al. (2020).

Fourier transform infrared spectroscopy (FTIR) is widely used for quantifying iCVD copolymer composition. Figure 3A displays the FTIR spectra for a series of copolymers grown from the monomers methacrylic acid (MAA) and ethylene glycol dimethacrylate (EGDMA) (Decandia et al., 2020). The most intense peak in Figure 3A, as well as in the FTIR spectra of many iCVD films, is the stretching mode of the carbonyl group (C=O). Fortuitously, the C=O stretching peak is located in a region of the FTIR spectrum that is virtually free of interference from peaks arising from other types of bonding environments. The C=O stretches appear around 1,700 cm^−1^, with its precise position varying from monomer to monomer. For example, the adsorption components centered at 1,702 and 1,730 cm^−1^ in Figure 3A are assigned to the MAA and EGDMA, respectively. Analyzing the areas of the components present in the C=O stretching region is often used to determine the ratio of monomeric units incorporated into the iCVD copolymers.

**Figure 4:**
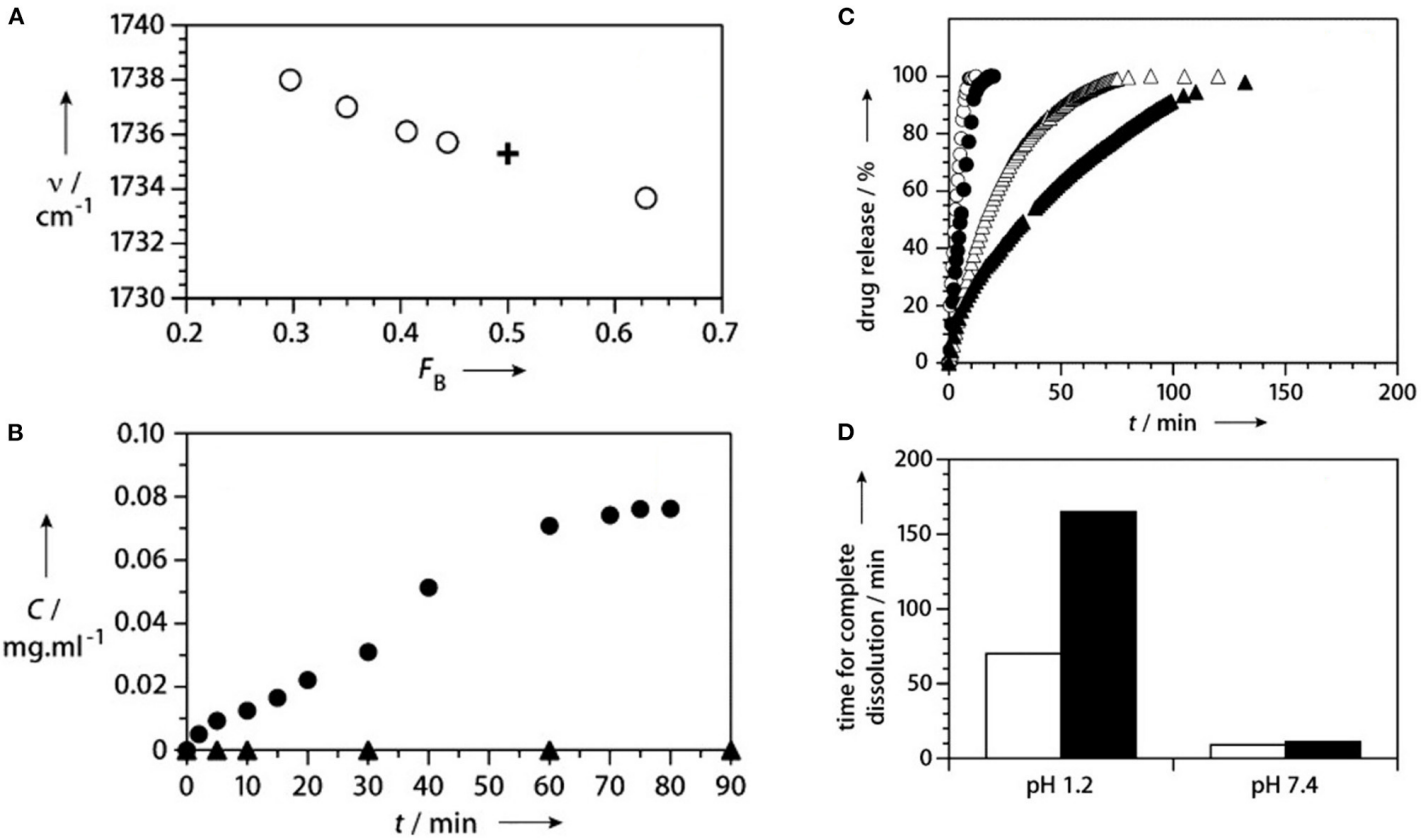
Composition and release kinetics for iCVD enteric coatings. **(A)** In the FTIR spectrum, the position of the carbonyl component for EA (open circles) shifts with F_B_, the fraction of EA in the P(MAA-co-EA). This shift confirms the iCVD copolymerization of the two monomers. For comparison, the cross shows the values for the commercial enteric coating, Eudrajit. For measurements at pH 1.2 (filled triangles) and pH 6.8 (filled circles): **(B)** release of fluorescein dye from a planar interface encapsulated with P(MAA-co-EA) and **(C)** release of ibuprofen from encapsulated microcrystals. The open triangles and circles and triangles show the data obtained without encapsulation at pH 1.2 and 7.4, respectively. **(D)** The time for complete dissolution of the ibuprofen is longer for the coated (black bars) than for uncoated (white bars) particles at the same pH and significant longer at pH 1.2 than at pH 7.4. Reprinted with permission from Lau and Gleason (2007).

The C=O stretch position has been used to confirm copolymerization as opposed to formation of a solid mixture of homopolymer chains (Lau and Gleason, 2007). For a series of iCVD copolymers produced using the monomers MAA and ethyl acrylate (EA), the central position of the EA component shifts monotonically with the composition (Figure 4A). This shift reflects the increasing probability that EA units bond to MAA monomer units. For EA homopolymer chain formation, the peak position would not shift.

**Figure 5:**
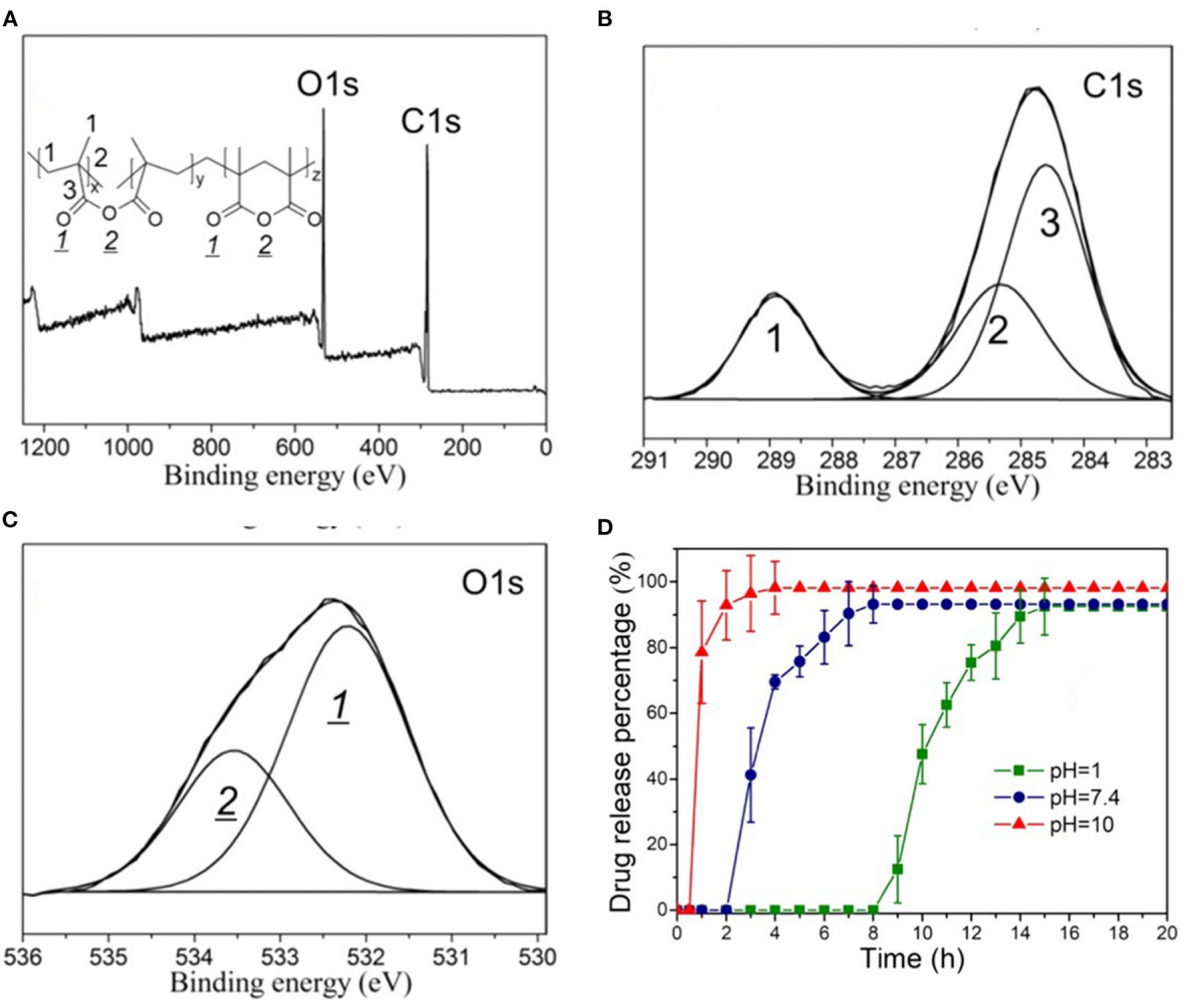
XPS spectra of the homopolymer PMAH: **(A)** survey scan and high-resolution scans for **(B)** C 1s, and **(C)** O 1s. In **(C)**, the ratio of the areas of the carbonyl oxygen and the ester oxygen is 2:1, which agrees with the stoichiometry of the structure shown in the insert of **(A)**. **(D)** The pH dependent release kinetics of rifampicin encapsulated by the biodegradable iCVD P(MAH-co-MAA). Reprinted with permission from Shi et al. (2018).

Another common characterization method used to obtain iCVD film composition is x-ray photoelectron spectroscopy (XPS). Figure 5 displays the use of XPS for the characterization of an iCVD homopolymers grown from MAH (Shi et al., 2018). The XPS survey scan (Figure 5A) show the presence of carbon and oxygen, as expected from the structure of the monomer and noting that hydrogen is not detected by XPS. The component peaks in the C 1s (Figure 5C) and O 1s (Figure 5D) high-resolution XPS spectra are labeled according to the chemical structure shown as the inset in Figure 5A. The two components observed in the O 1s spectra at 532.2 and 533.5 eV were assigned as oxygen in a carbonyl bond (>C=O) and oxygen in an ester bond (-C-O-C-), respectively. As expected from the stoichiometry of MAH, the observed peak area for the carbonyl oxygen in Figure 5C is twice that for the ester oxygen. Changes in the areas of the O 1s XPS spectra are valuable for quantifying changes in backbone structure. The concentration of the carbonyl oxygen relative to the ester oxygen increases as the MAH units undergoes biodegradation by hydrolysis of the ester group. The relative proportion of the carbonyl oxygen also increases when MAH is copolymerized with the carbonyl-containing monomer MAA. Thus, P(MAH-co-MAA) is of interest for release application because this copolymer degrades more rapidly than the homopolymer produced from MAH.

The XPS measurements are typically sensitive to the atomic fractions present in the top 10–20 nm of the film. In contrast, standard FTIR measurements provide the average chemical bonding configurations throughout the entire film thickness. When the FTIR and XPS results match, the composition of the bulk film and of the surface are the same. An example of excellent agreement between the FTIR and XPS compositional analyses was observed in a series of iCVD copolymers grown from systematically varied ratios of the monomer HEMA and the crosslinker ethylene glycol diacrylate (EGDA) (Chan and Gleason, 2006). When a compositional gradient exists between the top surface and the bulk film, angle resolved XPS can be used to obtain the composition in even narrower near surface regions (Yang and Gleason, 2012).

### Interfacial Grafting of iCVD Layers

Multiple grafting methods have been developed to form durable covalent bonds across the interface between the substrate and the iCVD layer (Gleason, 2015; Matsumura et al., 2019). Grafting greatly enhances durability by reducing the probability of delamination. For *ex-situ* grafting methods, the substrate is pretreated before being placed in the iCVD vacuum chamber. One *ex-situ* pretreatment for grafting begins with exposing a silicon wafer to an oxygen plasma. Next, the resulting plasma-activated surface is exposed to vinyl silane. The resulting vinyl terminated substrate is then placed inside the iCVD chamber. After pumping down the chamber to establish a vacuum, the iCVD process can proceed through the surface vinyl groups to yield grafted macromolecular chains. For *in situ* grafting methods, the substrate is pretreated inside of the iCVD vacuum chamber. Then without breaking vacuum, the conditions of the chamber are changed to begin iCVD film growth. One method for *in situ* grafting begins with pretreating the substrate using the *tert*-butyl peroxide (TBPO) initiator alone at filament temperatures >300°C. The thermal decomposition of the TBPO vapors produces *tert*-butoxy radicals, which further decompose to form methyl radicals. These methyl radicals can abstract a hydrogen atom from the surface, leaving behind a surface free radical as an active site for the growth of grafted polymers.

## Encapsulation by iCVD for Controlled Release

### Planar Configurations

**Figure 6:**
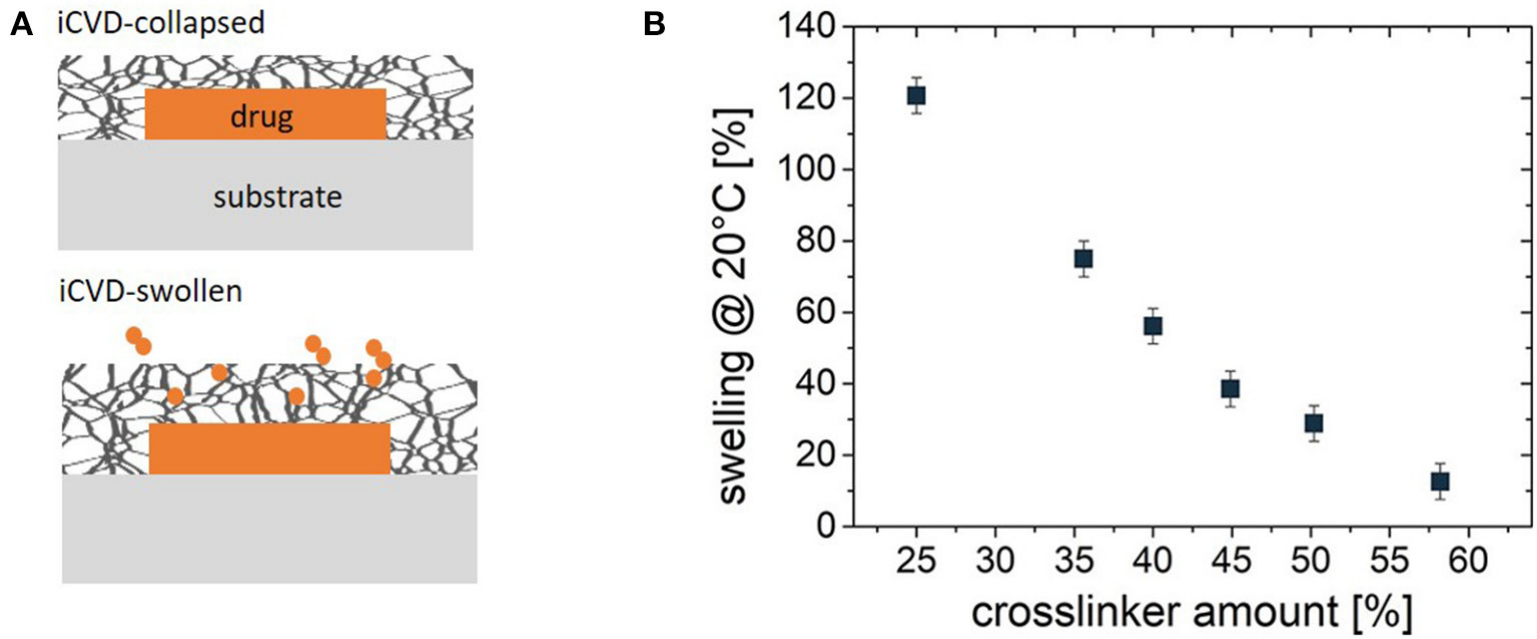
**(A)** Schematic of the planar bilayer configuration for encapsulation of a solid drug by a crosslinked iCVD hydrogel. At the top, the iCVD film is in its collapsed state and thus serves as a barrier against controlled release of the drug. At the bottom, swelling expands both the iCVD layer thickness and mesh size, permitting the diffusive release of the drug. **(B)** The % thickness change upon swelling for ~70 nm thick P(NIPAAm-co-DEGDVE) layers with different amounts (%) of the DEGDVE crosslinker in the film. Panel **(B)** reprinted with permission from Muralter et al. (2018).

Encapsulation of planar geometries allows quantification of the fundamental kinetics of drug release. These studies utilize a flat substrate, such as a glass slide. A solution containing the drug is either drop-cast or spun-cast onto the substrate (Christian et al., 2018; Decandia et al., 2020). The solvent evaporates, leaving behind a layer of the solid drug. For the encapsulation step, an iCVD film is grown directly onto the drug layer, creating a bilayer structure. The low substrate temperature for the iCVD process, typically room temperature, avoids damage or sublimation of the drug molecules. If the drug layer is applied to only partially cover the substrate, full coverage of the substrate by the iCVD layer will completely encapsulation of the drug layer's top and side surfaces (Figure 6A) (Christian et al., 2018). Coating down the side edges of the drug layer is essential for avoiding release from unmodified surfaces of the drug.

Drug release rates are often measured before and after of encapsulation. Reduced rates are typically seen after iCVD encapsulation (Figure 3B). The rate of drug release depends strongly on the iCVD layer thickness and composition. Figure 3B also shows that release rate decreases with an increased percentage of the crosslinker EGDMA in MAA-based copolymers. In aqueous environments, hydrophilic iCVD hydrogel layers can undergo significant swelling (Gleason, 2020a). Figure 3C show that 200 nm thick MAA-based iCVD films reach an equilibrium swelling thickness after a few minutes of immersion. Increasing the percentage of crosslinking lowers the thickness increase resulting from film swelling (Muralter et al., 2018) (Figure 6B). However, some level of crosslinking of the hydrogel is often essential to prevent complete dissolution of the film. Swelling increases a polymer's average mesh size, facilitating diffusion (Table 3). In addition, the diffusivity of the drug may also be impacted by hydrogen bonding and hydrophobic interactions between the drug and the hydrogel (Werzer et al., 2019).

For the first report of control release using iCVD layers, MAA and EGDA, were selected as the comonomers, with the goal of achieving enteric behavior (Lau and Gleason, 2007). Using planar bilayers of fluorescein dye encapsulated with a 400 nm thick iCVD P(MAA-co-EDGA) achieved enteric controlled release (Figure 4B) (Lau and Gleason, 2007). Without encapsulation, the solid dye dissolves away in <5 min at both pH 1.2 and 6.8. With encapsulation, no release of the dye occurs during soaking at pH 1.2 over the 90-min testing period. Raising the pH to 6.8 allows release of the dye over 60 min, ~5-fold slower than from the uncoated dye. The difference in release rates at the different pH levels corresponds to different degrees of swelling of the iCVD layers. Using spectroscopic ellipsometry to measure the increase film thickness at different pH values confirms there is minimal swelling (~5%) at low pH (≤5.0), while significant swelling (>30%) of the iCVD layer occurs at high pH values (≥ 6.5).

For the drug clotimiazole, two iCVD copolymers, P(HEMA-co-EGDMA) and P(MAA-co-EGDMA) were used as planar encapsulation layers (Table 2) (Christian et al., 2016). The clotimiazole was drop-cast onto glass, forming of a solid, amorphous drug layer. After 48 h in ambient conditions, the unencapsulated drug layer becomes predominately crystalline. In contrast, the drug remains predominately amorphous when encapsulated by iCVD, with only a few crystallites visible by microscopy. In their amorphous form, drugs dissolve more readily, as there is no enthalpy of crystallization acting as barrier to dissolution. Thus, preserving the amorphous phase is desirable for producing faster drug release rates. An analogous ability to alter the stable phase of underlying film has also be observed for iCVD films grown directly on top of block copolymers (Suh et al., 2017).

In another study utilizing the planar bilayer geometry, solid layers of the pain relief drug indomethacin were directly encapsulated by 200 nm thick iCVD layers (Christian et al., 2018). The kinetics of drug release were measured in pH 5.8 phosphate buffer solution at 25, 37, and 50°C. The observed rates depended dramatically on the composition of the iCVD permeation control layer. The slowest rate was observed using the crosslinked homopolymer PEGDMA at 25°C. The PEGDMA layer extended the release period to 20 h, far longer than the few minutes required for uncoated indomethacin to dissolve at the same conditions. The experimentally measured release kinetics for indomethacine at 37°C for iCVD P(HEMA-co-EGDMA) copolymers was pseudo-first order. The associated effective rate constant for release, k, was (0.39 ± 0.01) × 10^−3^ min^−1^ for the copolymer having 50% crosslinking. A more than 10-fold higher value of k, (5.58 ± 0.04) × 10^−3^ min^−1^ was measured for the copolymer having only 25% crosslinking. Measurements of k taken at three different temperatures were consistent with Arrhenius behavior. The release kinetics of the PHEMA could not obtained because immersion in aqueous solution caused this uncrosslinked homopolymer to detach from the underlying drug. This detachment may have occurred in order to relieve the interfacial stress produced by a high degree of swelling.

For the water soluble antibiotic, gentamicin, drug release kinetics were studied using planar bilayers (Decandia et al., 2020). For fabricating the drug layer, the gentamicin was spun-cast, as drop-casting resulted in much rougher films. Drug layers with smoother surfaces reduce the probability of pinhole formation in the subsequently grown iCVD encapsulation layer. The copolymer used for of the iCVD layer was P(MAA-co-EGDMA), which is a pH responsive hydrogel. The kinetics of gentamicin release in water at 37°C were obtained using ~200 nm thick encapsulation layers. The volume fraction of the EGDMA crosslinker incorporation into the encapsulation layer was systematically varied (Figure 3A). Higher crosslinker concentrations reduces the increase in thickness due to swelling (Figure 3B) and produces a corresponding reduction in the rate of drug release (Figure 3C). For uncoated gentamicin, k is 2.86 min^−1^. After encapsulation, k decreases to 0.06, 0.04, and 0.02 min^−1^ for the iCVD copolymers having EGDMA incorporation of 60, 70, and 80%, respectively. The gentamicin release kinetics were also analyzed using the model of Korsmeyer and Peppas. This model contains the assumption that the drug is embedded in the polymer layer rather than having a drug reservoir coated by a polymer layer. A reasonable statistic fit to the data was obtained and the extracted values for the fitting parameters were consistent with diffusive transport of the drug through the iCVD layer (Decandia et al., 2020).

Drug efficacy of the water-soluble antibiotic gentamicin was tested after release by diffusion through the iCVD encapsulation (Decandia et al., 2020). The released gentamicin was demonstrated to retain antibiotic activity against two strains of bacteria, Staphylococci aureus DSM799 (gram-positive) and Pseudomonas aeruginosa DSM939 (gram-negative). These experiments confirmed the stability of gentamicin during the all-dry, low-temperature process of iCVD encapsulation.

**Figure 7:**
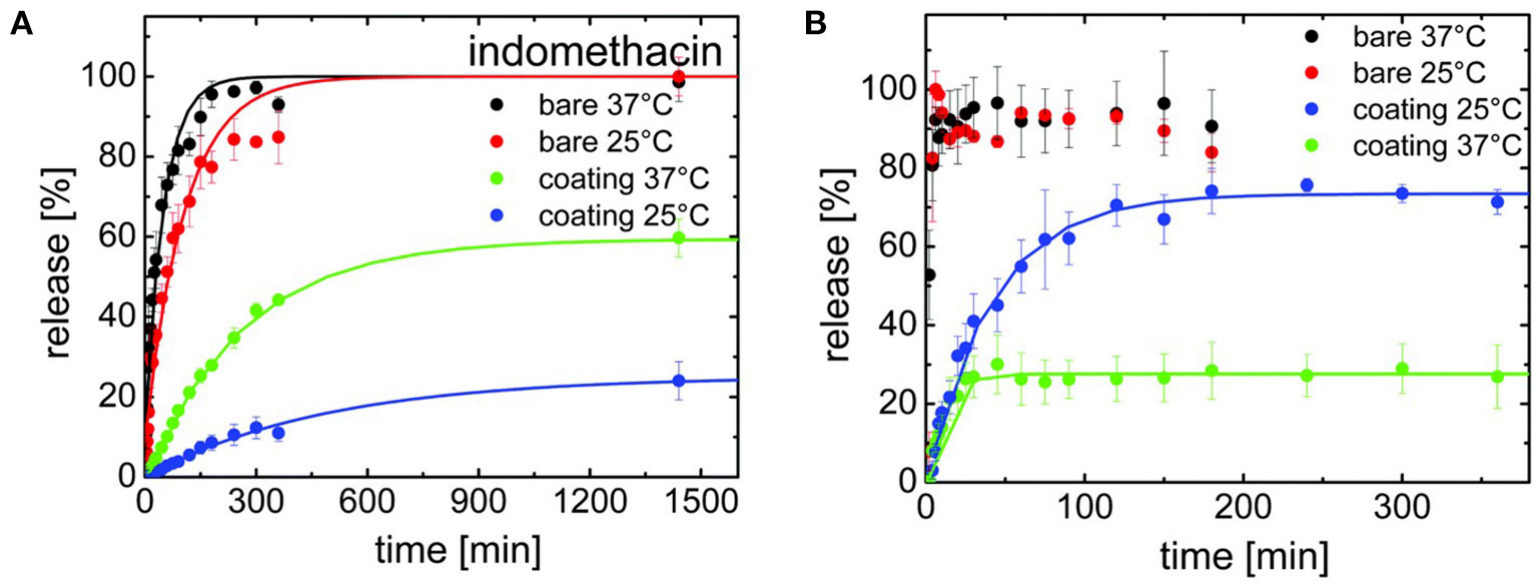
Release kinetics for indomethacin for both bare surfaces and for drug surfaces encapsulated with a thermoresponsive iCVD copolymer, (NIPAAm-co-DEGDVE). The LCST for the iCVD film lies between the measurement temperatures of 25 and 37°C. **(A)** At pH 3, the bare drug releases quickly at both temperatures. The iCVD coating provides a greater hindrance to release at 25°C than at 37°C. **(B)** At pH 7, fast release stills occurs for the bare drug at both temperatures. However, the iCVD coating now provides a greater hindrance to release at 37°C than at 25°C, which is the inverse of the behavior seen at pH 3. Reprinted with permission from Werzer et al. (2019).

Release kinetics were compared for three drugs encapsulated with iCVD poly(NIPAAm-co-DEGDVE) in the planar bilayer geometry (Werzer et al., 2019). The thickness of the iCVD layers was ~200 nm. Release rates were measured at temperatures both above and below the LCST (~29°C) of the thermoresponsive iCVD hydrogel. Below the LCST, the hydrogel is in an expanded state. Above the LCST, the hydrogel collapses, which was anticipated to slow the rate of drug release. Unexpectedly, the collapsed state exhibited higher release rates than the expanded state for measurements taken at pH 3 (Figure 7A). This surprising result was observed for the release of all three drugs: indomethacin, clotimazol, and phenytoin. To explain this counter-intuitive result, it was hypothesized that the drugs experience an additional hindrance to diffusion when the hydrogel is in its expanded state. The additional hindrance was ascribed to the formation of hydrogen bonds between the drug and hydrogel in the expanded state. At neutral pH, the indomethacin becomes deprotonated and is less likely to participate in hydrogen bonding with the hydrogel. At neutral pH, the expanded hydrogel state below the LCST exhibited a higher rate of indomethacin release than the collapsed state present above the LCST (Figure 7B). Thus, at neutral pH, indomethacin release rates follow the trend anticipated from the swelling behavior of the thermoresponsive hydrogel.

Growing thicker iCVD layers, allows the fabrication of free-standing membranes (Tufani and Ince, 2015). The free-standing geometry permits the quantitative determination of the diffusivity coefficients of the permeating species (Tufani and Ince, 2015). It is important to note that free-standing films can swell in three dimensions. In contrast, films pinned to a planar interface swell only in one dimension, which is orthogonal to the plane of the interface (Tenhaeff and Gleason, 2009). Thus, the same materials will exhibit a greater volumetric increase in a three-dimensional swelling geometry than for one-dimensional swelling. Thus, mesh size and the diffusivity of drugs will be higher for a free-standing membraned than for surface pinned layer of the same material (Table 3). To achieve a free-standing film, the iCVD growth proceeds directly on top of a sacrificial layer. The sacrificial layer consists of polyacrylic acid that had been spun-cast onto silicon wafers. Immersion in water causes the sacrificial layer to dissolve away, thus detaching the iCVD film.

Free-standing iCVD P(HEMA-co-EGDMA) copolymers were produced having either 0.32 or 0.47 as the fraction of the EGDMA crosslinker (Tufani and Ince, 2015). For both compositions, the dry thickness was 1,000 nm, the rms surface roughness was <5 nm and the layers remained intact with careful handling. Equilibration in water resulted in the free-standing layer with the higher crosslinker content to swell to a thicknesses of 1,660 nm, yielding a calculated average mesh size of 0.57 nm (Table 3). For the less crosslinked composition, swelling results in an equilibrium thickness of 1,820 nm and a calculated average mesh size of 0.65 nm. Thus, as expected, lowering the degree of crosslinking results in a higher degree of swelling and a larger mesh size. Control of the average mesh size in the iCVD hydrogel layer provides control over the release rates based on the hydrodynamic size of the diffusing species. At 25°C, a diffusion coefficient of 1.71 × 10^−7^ cm^2^/s was measured for alizarin yellow dye (molecular weight 309.21 g/mol) in the less crosslinked copolymer. The same measurement using the more crosslinked copolymer, having a corresponding lower mesh size, gave a lower diffusion coefficient by a factor of four.

### Conformal Encapsulation of Particles, Fibers, and Textiles

A range of iCVD process conditions can provide conformal coverage, in which films of uniform thickness form over complex shapes and geometric features. The conformal coverage of iCVD has been demonstrated at both the micro- and nano-scales (Moni et al., 2017). The iCVD method has the demonstrate ability to conformally coat individual micro- and nano- scale particles without aggregation (Lau and Gleason, 2006c) (Figure 2). Because no liquid phase is present during iCVD process, no surface-tension driven phenomenon can take place. In contrast, for wet encapsulation processes, such as spray drying, surface tension effects drive the formation of liquid bridges between particles. The bridges can cause the particles to bind together into aggregates during drying. The aggregation problem becomes more acute when the dimensions of the particle fall below 100 μm.

The first report of the application of iCVD for control release layers was demonstrated for ibuprofen, where the iCVD conformally encapsulated drug microcrystals 25 μm across (Figures 4C,D) (Lau and Gleason, 2007). The ability of iCVD to conformal coat microparticles was also explored for controlling the release of agrochemicals (Bose et al., 2012a). The iCVD encapsulation slowed the release from microparticles of a water-soluble crop protection compound (CPC). Slowing the release rate is desired for avoiding the CPC concentration reaching high, or even toxic, levels in the soil. The CPC particles, having a nominal size of only 3 μm, were successful encapsulated with conformal iCVD polymers. The two homopolymers were evaluated as encapsulants used the corresponding monomers glycidal methacrylate (GMA) and cyclohexylmethacrylate (CHMA). The iCVD encapsulant thicknesses ranged between 330 and 594 nm. The release of the CPC from iCVD encapsulated particles was monitored over the course of 80 h. The release rate as a function of time followed non-steady state Fickian diffusion. The diffusion coefficient of the CPC through the different types of iCVD layers tested fell in the range between 1.58 × 10^−7^ and 2.69 × 10^−7^ cm^2^/s.

Conformal iCVD encapsulation has also been applied to drug-loaded fibers by encapsulating of each individual fiber within a textile (Perrotta et al., 2018). Controlled drug release from textiles is of considerable practical interest for numerous applications including dressings that accelerate wound healing and for transdermal drug delivery (Ghasemi-Mobarakeh et al., 2019). Additionally, the encapsulation of biodegradable fiber-based substrates is desired for fabricating implantable patches utilized for site-specific drug delivery (Sayin et al., 2019b). Fiber-based substrates have a high surface area available for drug release. Extremely high surface area can be achieved using nanofiber mats, such as those produced by electrospinning (Sayin et al., 2019b).

The high surface areas of fiber-based substrates can be maintained when the encapsulation is conformal. The iCVD polymers can grow conformally around the circumference of each individual fiber without blocking the pores between the fibers. In contrast, surface area can be reduced by non-conformal encapsulation processes. For example, a film which bridges the open space between different fibers results in blockage of the pore structure within a fabric. Formation of the undesirable bridging films can be driven by surface-tension based phenomena present in liquid-phase coating processes (Heydari Gharahcheshmeh et al., 2020).

The iCVD conformal encapsulation process occurs at low growth surface temperatures and in the absence of solvents. These two characteristics of the iCVD process are compatible with the vast majority of fiber-based substrates and drugs. Thus, iCVD layers can be applied directly on top of drug-loaded fibers without damaging either the drug or the fibers.

Conformal iCVD encapsulation has been demonstrated for a variety of common fabrics including cotton and poly(ethylene terephalate) (PET) loaded with drugs (Ghasemi-Mobarakeh et al., 2019). Successful conformal iCVD encapsulation was also demonstrated on mats of nanofibers of different compositions including and polycaprolactone (Ghasemi-Mobarakeh et al., 2019), polyvinyl alcohol (Sayin et al., 2019b), and gelatin (Mansurnezhad et al., 2020). Drop-casting was one method utilized to load drugs onto the fabrics (Ghasemi-Mobarakeh et al., 2019). In other studies, the therapeutic agent was directly incorporated into the matrix of the fibers during the electrospinning process (Sayin et al., 2019b). The iCVD polymers used for encapsulation of the fiber-based substrates contained a hydrogel-forming monomer (Gleason, 2020a). Copolymerization with a crosslinking monomer typically enhances the stability of the iCVD hydrogel layers. The degree of crosslinker incorporation provides a means to optimize the kinetics of controlled release through systematic variation of the mesh size displayed by the iCVD hydrogels.

**Figure 8:**
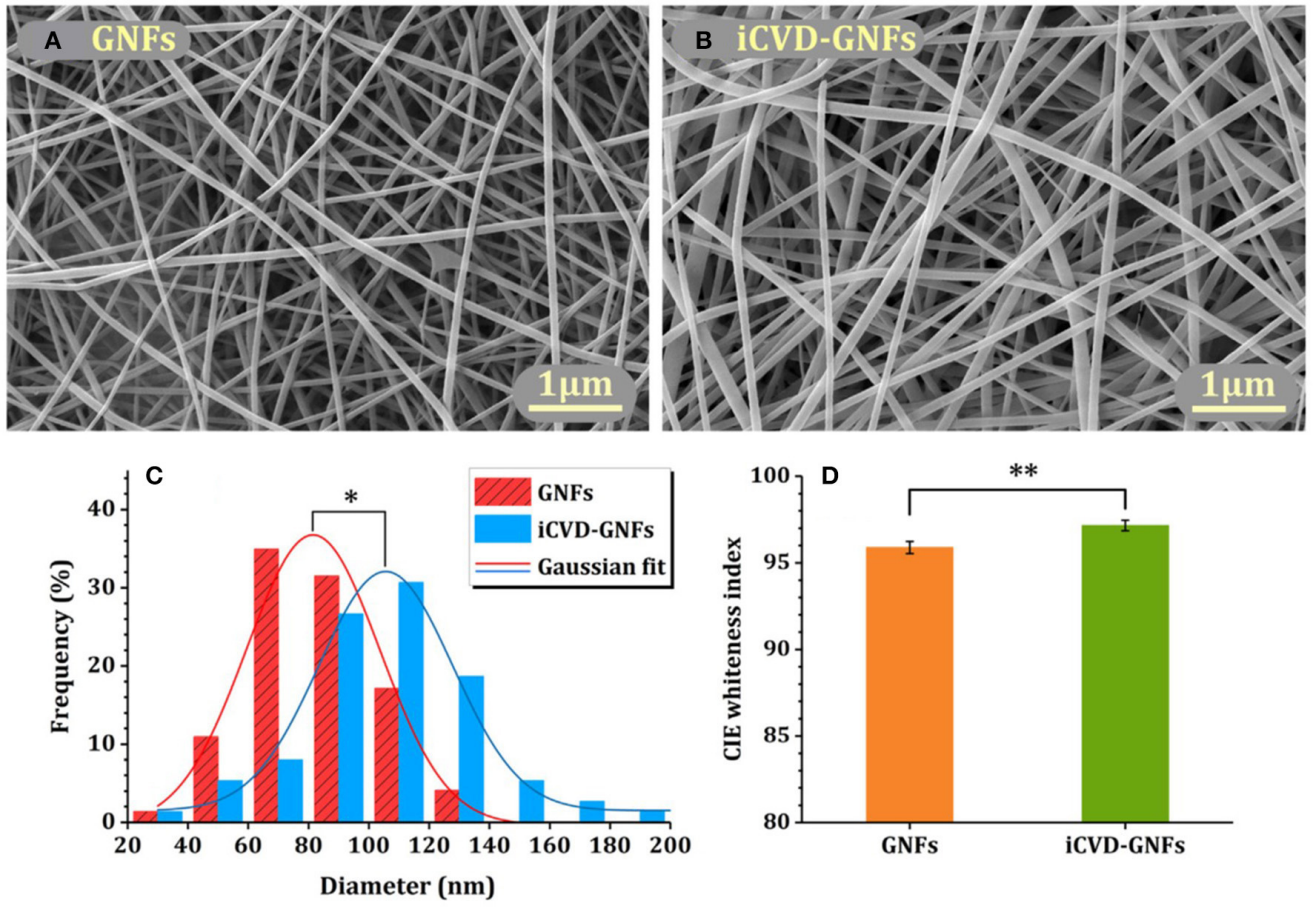
Electrospun gelatin nanofibers (GNFs) **(A)** bare and **(B)** after conformal coating by crosslinked iCVD homopolymer P(EGDMA). Image analysis shows that **(C)** the diameters and **(D)** the whiteness of the coated GNFs have increased as compared to the bare substrate. Reprinted with permission from Mansurnezhad et al. (2020). Statistically significant differences *p ≤ 0.005 and **p ≤ 0.05.

Homopolymerization of crosslinking monomers, such EGDMA, leads to low average mesh size (Table 2) causing these films to act a diffusion barrier. Indeed, conformal coatings of iCVD P(EGDMA) were able to protect the underlying electrospun gelatin nanofibers (GNFs) from dissolution in aqueous solution during a 31 day long observation period (Mansurnezhad et al., 2020). Without coating, the dissolution of the GNFs is essentially instantaneous. The ability to greatly slow dissolution confirms that the iCVD coatings are conformal forming around the nanofibers. SEM analysis of the GNFs before and after surface modification, Figures 8A,B, respectively, also verifies the conformal nature of the iCVD process. Statistical analysis of the SEM micrographs provides average fiber diameters of 83.4 ± 30 nm before coating, and 106.6 ± 30 nm after coating (Figure 8C). Thus, the average iCVD coating thickness on the fibers is 11.6 nm. The coated fibers also have a statistically whiter appearance (Figure 8D). Evaluating the surface morphology of the coated fibers by 2D atomic force microscopy (AFM) shows <2 nm of root mean square roughness.

**Figure 9:**
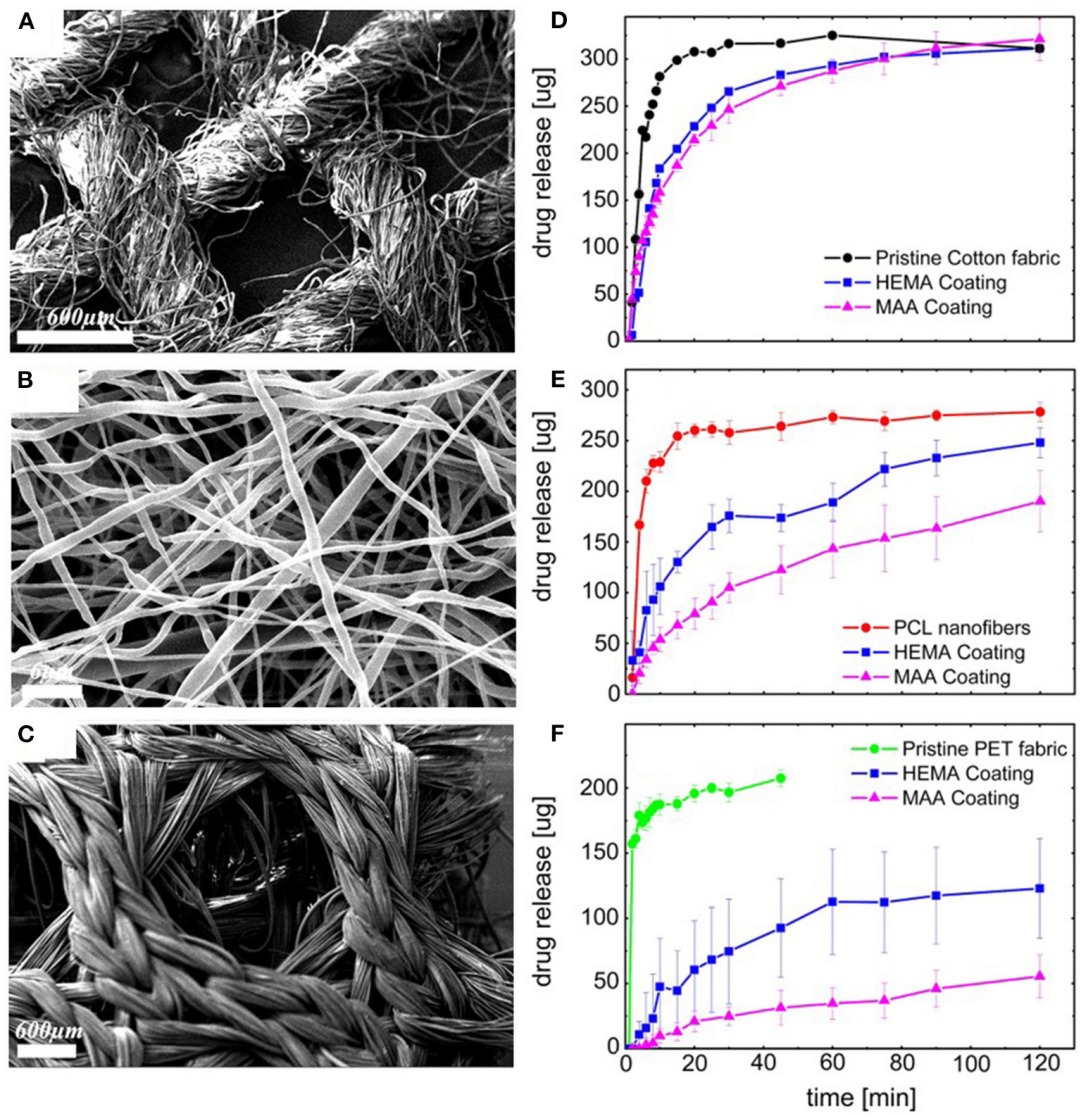
Scanning Electron Micrographs (SEM) of conformal coatings of iCVD P(HEMA-co-EGDMA) on clotrimazole loaded fiber-based substrates: **(A)** cotton gauze, **(B)** PCL electrospun nanofiber mats, and **(C)** warp-knitted spacer PET fabric. The corresponding release kinetics are given in **(D**–**F)**, respectively. Each of the release kinetic panels also shows data for the pristine fabrics and fabrics conformally encapsulated with PMAA and PEGDMA. Reprinted with permission from Ghasemi-Mobarakeh et al. (2019).

Controlled release from fiber-based substrates was achieved by loading the fibers with the drug clotrimazole, followed by conformal encapsulation using iCVD (Ghasemi-Mobarakeh et al., 2019). Cotton gauze, warp-knitted spacer poly(ethylene terephalate) (PET) fabric, and electrospun polycaprolactone (PCL) nanofiber mats were used as substrates. Figures 9A–C displays SEM micrographs of the substrates after coating with iCVD P(HEMA-co-EDGMA). Shown in the original paper, but not reproduced here, are SEM micrographs of all three types substrates prior to coating and after coating with a different iCVD polymer, P(MAA-co-EDGMA). The similarity of the before and after images for all three substrate types confirms the conformal nature of the processes used for obtaining both iCVD compositions.

Figures 9D–F displays the drug release from the fiber-based substrates. For all three types of substrates, the drug-loaded pristine fibers give the fastest release (Ghasemi-Mobarakeh et al., 2019). The iCVD layers are observed to act as barrier against the initial sudden release of the drug and also slow the kinetics of drug release at longer times. The greatest hindrance to release and lowest fraction of overall drug release is observed on the PET fabric. The smallest difference in release rates and highest fraction the overall drug released occurs with the cotton gauze substrates. Additionally, for all three substrates, the HEMA-based neutral hydrogel coating gives faster release than the MAA-based pH-responsive hydrogel coating. The observation of the higher release rates from the HEMA-based coatings, reflect differences in multiple factors, including coating thickness, mesh size, and drug-hydrogel interactions.

Fibers electrospun from a mixture of the biodegradable polymer polyvinylalcohol (PVA) and Rose Bengal (RB) were successful encapsulated by iCVD. The RB is chemotherapeutic agent for brain cancer. The encapsulation layer was characterized by SEM, FTIR, and XPS (Sayin et al., 2019b). As was also the case for other types of electrospun fibers (Figures 8A,B, 9C), SEM micrographs (not reproduced here) confirmed that the iCVD coating was conformal. The pH responsive p(4VP-co-EGDMA) crosslinked copolymer formed concentrically around each fiber without forming bridging film defects between different fibers. Thus, the encapsulated mat retained the high surface area of the underlying substrate. Image analysis the SEM micrographs before and after encapsulation determined that thickness of the as-deposited iCVD layer surrounding each individual fiber was 65 ± 5 nm. The presence of the iCVD layers was also confirmed by chemical characterizing using FTIR and XPS. The FTIR spectra identified pyridine ring vibrations only after encapsulation. The pyridine structure is only present in the 4VP moieties contained in the iCVD film. In the XPS survey spectra, the N 1s absorption appears only after encapsulation, which corresponds to the presence of nitrogen only in the 4VP units of the iCVD copolymer.

The pH responsive behavior of the iCVD p(4VP-co-EGDMA) was quantified from the swelling experiments performed using the planar bilayer geometry (Sayin et al., 2019b). The p(4VP-co-EGDMA) film swelled by 62 ± 10 % at pH 4, but only swelled <5% at pH 6.5 or 9. The average mesh size at low pH was calculated to be 1.16 nm at low pH, decreasing to 0.69 nm at higher pHs. The greater swelling in acid solution was attributed to the deprotonation of the 4VP unit. The hydrodynamic radius of RB is 1.28 nm.

The iCVD encapsulation reduced the release rate of RB and enabled pH responsive controlled release of RB from the electrospun fiber mat (Sayin et al., 2019b). The iCVD process for conformal encapsulation by p(4VP-co-EGDMA) damaged neither the nanofibers nor the therapeutic agent. To confirm that the drug retained its activity after being exposed to the iCVD process, RB released from iCVD encapsulated nanofiber mat was shown to produce an anticancer response in tests utilizing glioblastoma multiform cancer cells (U87MG).

### iCVD Encapsulation of Porous Media

Porous media can offer a high surface area for drug loading to create drug reservoirs of large volume. By selecting the appropriate processing conditions, iCVD polymers can deposit as capping layers over porous surface. Optimizing the composition and thickness of the iCVD capping layers produces the desired release characteristics.

**Figure 10:**
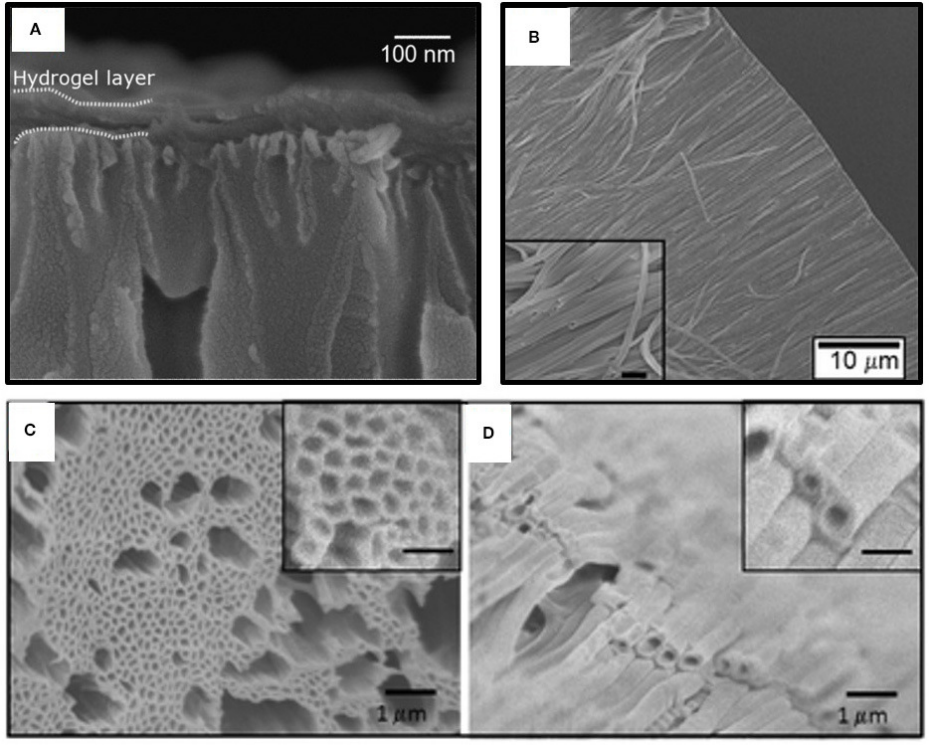
Scanning Electron Micrographs (SEM) of anodized aluminum oxide membranes (AAO) coated with iCVD hydrogel layers. **(A)** A non-conformal blanket layer formed across the top AAO surface. Arrays of iCVD hydrogel nanotubes formed by conformally coating the interior pore walls of the AAO and then selectively dissolving away the AAO template: **(B)** cross-sectional view of a dry array; **(C)** top-down view of a dry array; and **(D)** a swollen array resulting from loading the nanotubes with dye. For the insets in both **(C,D)**, the scale bar is 500 nm. Panel **(A)** reprinted with permission from Tenhaeff and Gleason (2009); panel **(B)** reprinted with permission from Ozaydin-Ince et al. (2011); and panels **(C,D)** reprinted from permission from Ozaydin Ince et al. (2010).

Thin iCVD layers can blanket over porous media when the iCVD processing conditions, such as high SR, favor non-conformal growth. Such blanket iCVD layer represent a permselective skin which is mechanically supported by bulk porous material. The advantages of forming the skins by iCVD include the ability to produce pinhole free layers <10 nm in thickness and the ability to use rapid *in situ* grafting strategies to anchor the permselective layer to the underlying mechanical support (Wang et al., 2017b). Indeed, iCVD was successful able to create supported membranes with pH responsive skins to control the passage of organic molecules, including the sugar glucose and the protein, bovine serum albumin (BSA) (Tenhaeff and Gleason, 2009). The iCVD permselective films spanned over the pores of an anodized aluminum oxide (AAO) support. Figure 10A displays a ~73 nm thick iCVD blanketing layer on top of an AAO membrane. Crosslinking by diethylene glycol divinyl ether (DEGDVE) and grafting increase the stability of the iCVD skin layer. In addition to the DEGDVE crosslinking monomer, two other monomers, MAH and N,N-dimethyacrylamide, were used to synthesize the iCVD terpolymer skin layer. The terpolymer is an ionic hydrogel which can swell to a much greater degree than neutral hydrogels, such as those based on the HEMA monomer. At pH 7, the terpolymer thickness was able to increase by >10,000% due to swelling. The corresponding mesh size was 3.2 nm. This mesh size allow the ready passage of glucose, while excluding BSA. De-swelling occurred when the external pH was switched to pH values <2.8.

Capping iCVD layers formed over oxidized porous silicon (pSi-Ox) have been explored for drug release applications (McInnes et al., 2012, 2016). The pSi-Ox is a biodegradable high surface area substrate (100–800 m^2^/g) and provides large volume reservoir for the drug. The pSi-Ox was produced by electrochemical etching of a silicon wafer in hydrofluoric acid, followed by a thermal oxidation in a tube furnace. Fluorescence spectroscopy was employed to monitor the release kinetics of the drug camptothecin (CPT) from bare and capped pSi-Ox over a 17 h period. Release rates were determined for both the initial burst period (~1–2 h) and for the subsequent linear release period. The data was analyzed using common models including zero-order, first-order, Higuchi, Hixon-Crowell, and Ritger-Peppas. The Higuchi model was found to provide the best statistical description of the data for all the uncoated and iCVD capped pSi-Ox samples.

Enteric release was observed when the crosslinked iCVD copolymer, poly(MAA-co-EDGA), formed an 340 nm thick cap over over the pSi-Ox (McInnes et al., 2012). The uncoated pSi-Ox does not display pH responsive behavior as confirmed by the similar kinetics observed for the release of CPT at pH 1.8 and 7.4. Significant slower release occurs after the pSi-Ox capped with iCVD poly(MAA-co-EDGA. Additionally, the capping achieves enteric behavior since the linear release rate (0.12 nmol/cm^2^ h) at pH 1.2 is lower than that at pH 7.4 (0.52 nmol/cm^2^ h). The change in rate can be attributed to the MAA-containing iCVD polymer having a limited swelling at low pH, but significant swelling with water at higher pH. The larger degree of swelling at high pH increases the diffusion rate of drugs through the iCVD layer, which leads to the higher release rate.

Temperature responsive drug release was achieved from pSi-Ox using a capping layer fabricated using a different crosslinked iCVD copolymer, p(NIPAAm-co-DEGDVE) (McInnes et al., 2016). The uncoated pSi-Ox gave similar burst release rates of CPT (~20 nmol/cm^2^ h) at both 25 and 37°C. Adding the thermoresponsive iCVD hydrogel capping layer increases the barrier to drug release. At 25°C, which is below the LCST of the iCVD layer, the burst release rate is reduced to 3.4 nmol/cm^2^ h. At body temperature, 37°C, which is above the LCST, the burst release rate is 4.8 nmol/cm^2^ h, which is higher than at 25°C but still substantially lower than for uncoated pSi-Ox.

The iCVD method has also been used to synthesize capping layers displaying pH responsive erosion (Shi et al., 2018). Upon erosion, the drug is exposed directly to aqueous solution, which can produce higher rates of drug release as compared to the rates achieved by requiring diffusion of the drug through an iCVD capping layer. Copolymerization of MAH with MAA increases the rate of degradation of the iCVD capping layer (Shi et al., 2018). The degradation kinetics of iCVD pMAH and p(MAH-co-MAA) were observed to be pH responsive, with dramatically higher rates observed in alkaline and neutral environments than in acidic solutions (Figure 5). For these studies, microporous polylactide membranes served as the drug-carrying reservoir. The polylactide support also biodegrades, but at a slower rate than the iCVD capping layer. A common antibiotic drug, rifampicin, was loaded into the polylactide membranes prior to the iCVD capping step. Cross-sectional SEM micrographs show the capping layer thickness is ~1 μm.

### Polymeric Micro and Nano- Structures

The increased ratio of surface area to volume provided by micro- and nano-structures offers increased capacity for loading therapeutic molecules (Sayin et al., 2019a). Applications requiring the controlled delivery of biomolecules, motivates the development of polymeric nanostructures which are biocompatible. Stimuli-responsive behavior is also desired for externally controlling and triggering of drug delivery.

Nanotubes demonstrating externally actuated responsive release have been fabricating by growing iCVD polymers on the inside of templates (Ozaydin-Ince et al., 2011; Armagan and Ozaydin Ince, 2015). For templating nanostructures, iCVD growth conditions used (low SR) typically result in conformal coverage. With conformal iCVD conditions, a cylindrical layer of polymer forms on the interior walls of the template. In the case of cylindrical pores, the pore remains open but has a reduced diameter as compared to the unmodified template. The fabrication of co-axial bilayer nanotubes is readily achieved by carrying out the conformal deposition of a different iCVD polymer following the first iCVD step. Template removal releases the nanotubes.

A frequently used template is anodized aluminum oxide (AAO) membranes. The desirable features AAO templates include the cylindrical nature of its pores and ability to etch away the AAO by immersion in a hydrochloric acid solution. Figure 10B show an array of poly(HEMA-co-EGDA) nanotubes released from an AAO template (Ozaydin Ince et al., 2010). Using low SR (between 0.05 and 0.22) for the iCVD growth provides conformal coverage of the interior surfaces down the entire length of the pores. This conformal coverage is impressive, as the pores are 60 μm in length and 200 nm in diameter, corresponding to an aspect ratio of 300:1. The conformal iCVD growth shown in Figure 10B is a dramatic contrast to the non-conformal blanket coverage displayed Figure 10A. This difference in the degree of conformality results from varying the SR of the iCVD process utilized for growing the polymers over the AAO membranes.

The activated release of fluorescent dye (fluorescein-5-thiosemicarbazide, FTSC) was demonstrated using coaxial bilayer polymeric nanotubes (Ozaydin-Ince et al., 2011). The outer layer of the nanotubes is ~20 nm thick layer of thermally-responsive shape memory iCVD copolymer. This outer shape memory layer was synthesized from the monomer tert-butyl acrylate and the crosslinking monomer DEGDVE. The inner layer is an ~40 nm thick iCVD neutral hydrogel layer synthesized the monomer HEMA and crosslinking monomer EGDA. Figure 10C reveals that the after being released from their AAO template, the nanotubes are 200 nm in diameter with a wall thickness of 60 nm. The SEM micrograph does not provide sufficient contrast to resolve the two different iCVD layers which make up the nanotube wall. Loading with the FTSC dye swells the nanotube diameters to ~350 nm and the wall thicknesses to ~100 nm (Figure 10D). Raising the external temperature to 80°C causes the outer layer shape memory to shrink, activating burst release of the dye.

Thermoresponsive nanotubes were also fabricated utilizing iCVD poly(NIPAAm-co-EGDMA) (Armagan and Ozaydin Ince, 2015). This NIPAAm-copolymer was employed both in single wall nanotubes and as the outer layer of coaxial nanotubes. The inner layers of the coaxial nanotubes were either the neutral hydrogel poly(HEMA-co-EGDMA) or the pH responsive hydrogel poly(MAA-co-EGDMA). The overall wall thickness for all the nanotubes reported by this work fell in the range of 30–50 nm. Release of phloroglucinol dye was triggered by raising the temperature to 40°C. This temperature exceeds the LCST of NIPAAm (~32°C), causing the NIPAAm-containing walls to shrink and dye to be forced out. The single wall NIPAAm-based nanotubes provided the highest rate of release. Introducing the HEMA-based or MAA-based inner layers slows the release rate, thus tuning the response. Employing the hydrophilic HEMA-based inner walls resulted in the slowest release rate of the hydrophilic dye. Repeating of the release rate experiments multiple times using the same sample confirmed the stability of the iCVD nanotubes.

Bilayers structures, where the top layer was grown by iCVD, have been utilized to create self-wrinkling surfaces and self-bending microstrips (Oh et al., 2016; Muralter et al., 2019). The strong adhesion of the iCVD layer to underlying substrate creates pinning at the interface, producing the residual stress required for the self-responsive phenomena. The iCVD layer can also impart smart behavior, such as thermoresponsiveness or pH responsiveness.

**Figure 11:**
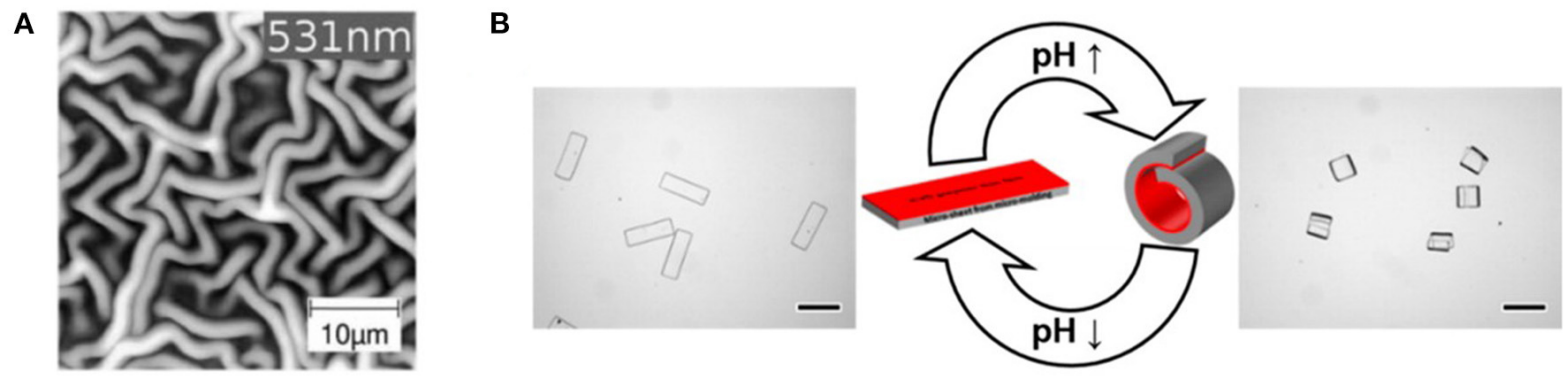
Responsive behavior of bilayers fabricated by depositing an iCVD layer on top of thicker conventional polymer substrate. The pinned interface between the iCVD layer and the substrate leads to **(A)** the formation of wrinkling patterns and **(B)** pH-responsive bending behavior. Panel **(A)** reprinted with permission from Muralter et al. (2019) and panel **(B)** reprinted with permission from Oh et al. (2016).

Wrinkled surfaces increase in the surface area available for drug release (Muralter et al., 2019). The wrinkled microstructures displayed in Figure 11A were created by growing an iCVD layer directly on top of a conventional enteric controlled drug release layer, Eudrajit. No prestrain was applied to the Eudrajit prior to the iCVD growth. This is unusual, as typically the formation of wrinkles requires prestraining of the bottom layer before the application of the top layer (Yin et al., 2012). The iCVD layer was synthesized from monomers N-vinylcaprolatam (NVCL) and the crosslinking monomer DEGDVE. The NVCL units impart thermoresponsiveness to the iCVD copolymer. Thus, the bilayer structure is multifunctional, having a thermoresponsive hydrogel layered on top of a pH-responsive hydrogel.

The ability to form smart 3D microstructures is anticipated to have applications to drug delivery (Oh et al., 2016). To demonstrate self-bending 3D microstructures, Janus microstrips were fabricated by depositing an iCVD layer on top of micromolded conventional hydrogel layer. The iCVD layer was a heavy crosslinked, mechanically tough, and passive layer synthesized, 100 nm thick, by the homopolymerization of divinylbenzene. The bottom layer consisted of terpolymer of HEMA, acrylic acid, and a crosslinking agent. The protonation and deprotonation of the acrylic acid units in the conventional hydrogel bottom layer are responsible for the pH responsive behavior of the microstrip. The resulting microstrip folded and unfolded in response to repeated cycling of the external pH (Figure 11B).

## Conclusions and Future Directions

The iCVD process enables the synthesis of a wide range of hydrogel polymer thin films of interest as encapsulation layers for controlled release. The iCVD process is compatible with a wide range of monomers and successfully retains the organic functional groups present in these monomers. Selecting and retaining the functional groups is essential for optimizing film properties, such as pH-responsive or temperature-responsive swelling. Additionally, systematic control of crosslinking incorporation enables optimization of the permeation properties of the iCVD encapsulation layers. The mild conditions of the iCVD process avoid damaging the drug which is being encapsulated. Reactor chambers for the iCVD process can be customized to coat non-planar substrates, such as microparticles. Large iCVD chambers have also be demonstrated.

The CVD process enables controlled release layers to form conformally over the complex geometries displayed by substrates such as microparticles, textiles, nano-fiber mats, and membranes. The conformality of the iCVD process can also be exploited for fabricating polymeric nanostructures. These non-planar geometries increase the surface area available for controlled release. Successful triggering of controlled release by changing the external temperature or pH have been demonstrated from these complex geometries by utilizing conformal iCVD polymer encapsulation layers. The iCVD layers can be either permselective or biodegradable.

Additional characteristics and compositions of iCVD polymers that have been demonstrated for other applications (Gleason, 2020b), and in particular for the permselective layers of membranes (Zhao and Gleason, 2020), may find future utility for controlled release applications. For example, photoresponsive iCVD polymers have been produced through the incorporation of chromophores, such as azobenzene (Unger et al., 2017) and styrene diazocine (Burk et al., 2020). Additional examples are other types of surface functionalization, such as by zwitterionic moieties (Yang et al., 2011; Yang and Gleason, 2012), and the production of compositional gradients as a function film depth (Montero et al., 2009; Schroder et al., 2020).

## Author Contributions

The author confirms being the sole contributor of this work and has approved it for publication.

## Conflict of Interest

KG is a co-founder of two companies commercializing iCVD polymers, GVD Corporation and DropWise Corporation.

## References

[B1] Alf M. E. Godfrin P. D. Hatton T. A. Gleason K. K. (2010). Sharp hydrophilicity switching and conformality on nanostructured surfaces prepared via initiated chemical vapor deposition (iCVD) of a novel thermally responsive copolymer. Macromol. Rapid Commun. 31, 2166–2172. 10.1002/marc.201000452 21567647

[B2] Alf M. E. Hatton T. A. Gleason K. K. (2011). Insights into thin, thermally responsive polymer layers through quartz crystal microbalance with dissipation. Langmuir 27, 10691–10698. 10.1021/la201935r 21806008

[B3] Armagan E. Ozaydin Ince G. (2015). Coaxial nanotubes of stimuli responsive polymers with tunable release kinetics. Soft Matter 11, 8069–8075. 10.1039/C5SM01074H 26333009

[B4] Bhat S. V. (2002). Cardiovascular implants and extracorporeal devices, in Biomacromolecules (Dordrecht: Springer), 130–162. 10.1007/978-94-010-0328-5_9

[B5] Boscher N. D. Wang M. Perrotta A. Heinze K. Creatore M. Gleason K. K. (2016). Metal–organic covalent network chemical vapor deposition for gas separation. Adv. Mater. 28, 7479–7485. 10.1002/adma.201601010 27296896

[B6] Bose R. K. Heming A. M. Lau K. K. S. (2012a). Microencapsulation of a crop protection compound by initiated chemical vapor deposition. Macromol. Rapid Commun. 33, 1375–1380. 10.1002/marc.201200214 22573697

[B7] Bose R. K. Nejati S. Stufflet D. R. Lau K. K. S. (2012b). Graft polymerization of anti-fouling PEO surfaces by liquid-free initiated chemical vapor deposition. Macromolecules 45, 6915–6922. 10.1021/ma301234z

[B8] Burk M. H. Schröder S. Moormann W. Langbehn D. Strunskus T. Rehders S. (2020). Fabrication of diazocine-based photochromic organic thin films via initiated chemical vapor deposition. Macromolecules 53, 1164–1170. 10.1021/acs.macromol.9b02443

[B9] Chan K. Gleason K. K. (2005). Photoinitiated chemical vapor deposition of polymeric thin films using a volatile photoinitiator. Langmuir 21, 11773–1711779. 10.1021/la051469g 16316113

[B10] Chan K. Gleason K. K. (2006). A mechanistic study of initiated chemical vapor deposition of polymers: analyses of deposition rate and molecular weight. Macromolecules 39, 3890–3894. 10.1021/ma051776t

[B11] Cheng C. Gupta M. (2018). Roll-to-roll surface modification of cellulose paper via initiated chemical vapor deposition. Ind. Eng. Chem. Res. 57, 11675–11680. 10.1021/acs.iecr.8b03030

[B12] Christian P. Ehmann H. M. A. Coclite A. M. Werzer O. (2016). Polymer encapsulation of an amorphous pharmaceutical by initiated chemical vapor deposition for enhanced stability. ACS Appl. Mater. Interfaces 8, 21177–21184. 10.1021/acsami.6b06015 27467099 PMC4999961

[B13] Christian P. Tumphart S. Ehmann H. M. A. Riegler H. Coclite A. M. Werzer O. (2018). Controlling indomethacin release through vapor-phase deposited hydrogel films by adjusting the cross-linker density. Sci. Rep. 8:7134. 10.1038/s41598-018-24238-w 29739950 PMC5940858

[B14] Coclite A. M. (2013). Smart surfaces by initiated chemical vapor deposition. Surf. Innov. 1, 6–14. 10.1680/si.12.00019

[B15] Decandia G. Palumbo F. Treglia A. Armenise V. Favia P. Baruzzi F.. (2020). Initiated chemical vapor deposition of crosslinked organic coatings for controlling gentamicin delivery. Pharmaceutics 12:213. 10.3390/pharmaceutics12030213 32121608 PMC7150873

[B16] Donadt T. B. Yang R. (2020). Vapor-deposited biointerfaces and bacteria: an evolving conversation. ACS Biomater. Sci. Eng. 6, 182–197. 10.1021/acsbiomaterials.9b01496 33305000 PMC7725245

[B17] Gao Y. Cole B. Tenhaeff W. E. (2018). Chemical vapor deposition of polymer thin films using cationic initiation. Macromol. Mater. Eng. 303:1700425 10.1002/mame.201700425

[B18] Ghasemi-Mobarakeh L. Werzer O. Keimel R. Kolahreez D. Hadley P. Coclite A. M. (2019). Manipulating drug release from tridimensional porous substrates coated by initiated chemical vapor deposition. J. Appl. Polym. Sci. 136:47858 10.1002/app.47858

[B19] Gleason K. K. (2015). CVD Polymers: Fabrication of Organic Surfaces and Devices. Weinheim: Wiley 10.1002/9783527690275

[B20] Gleason K. K. (2019). Organic surface functionalization by initiated CVD (iCVD), in Surface Modification of Polymers, eds Pinson J. Thiry D. (New York, NY: Wiley), 107–134. 10.1002/9783527819249.ch4

[B21] Gleason K. K. (2020a). Chemically vapor deposited polymer nanolayers for rapid and controlled permeation of molecules and ions. J. Vac. Sci. Technol. A 38:020801 10.1116/1.5132851

[B22] Gleason K. K. (2020b). Nanoscale control by chemically vapour-deposited polymers. Nat. Rev. Phys. 2, 347–364. 10.1038/s42254-020-0192-628346456

[B23] Gupta M. Gleason K. K. (2006). Large-scale initiated chemical vapor deposition of poly(glycidyl methacrylate) thin films. Thin Solid Films 515, 1579–1584. 10.1016/j.tsf.2006.05.021

[B24] Hanak B. W. Hsieh C. Y. Donaldson W. Browd S. R. Lau K. K. S. Shain W. (2018). Reduced cell attachment to poly(2-hydroxyethyl methacrylate)-coated ventricular catheters *in vitro*. J. Biomed. Mater. Res. Part B Appl. Biomater. 106, 1268–1279. 10.1002/jbm.b.33915 28631360 PMC5738300

[B25] Heydari Gharahcheshmeh M. Wan C. T.-C. Gandomi Y. A. Greco K. V. Forner-Cuenca A. Chaing Y.-M. (2020). Ultrathin conformal oCVD PEDOT coatings on carbon electrodes enable improved performance of redox flow batteries. Adv. Mater. Interfaces 7:2000855 10.1002/admi.202000855

[B26] Kovacik P. Del Hierro G. Livernois W. Gleason K. K. (2015). Scale-up of oCVD: large-area conductive polymer thin films for next-generation electronics. Mater. Horizons 2, 221–227. 10.1039/C4MH00222A

[B27] Lau K. K. S. Gleason K. K. (2006b). Initiated chemical vapor deposition (iCVD) of poly(alkyl acrylates): an experimental study. Macromolecules 39, 3688–3694. 10.1021/ma0601619

[B28] Lau K. K. S. Gleason K. K. (2006c). Particle surface design using an all-dry encapsulation method. Adv. Mater. 18, 1972–1701977. 10.1002/adma.200600896

[B29] Lau K. K. S. Gleason K. K. (2007). All-dry synthesis and coating of methacrylic acid copolymers for controlled release. Macromol. Biosci. 7, 429–434. 10.1002/mabi.200700017 17429803

[B30] Lau K. K. S. S. Gleason K. K. (2006a). Initiated chemical vapor deposition (iCVD) of poly(alkyl acrylates): a kinetic model. Macromolecules 39, 3695–3703. 10.1021/ma0601621

[B31] Loyer F. Bulou S. Choquet P. Boscher N. D. (2018). Pulsed plasma initiated chemical vapor deposition (PiCVD) of polymer layers – a kinetic model for the description of gas phase to surface interactions in pulsed plasma discharges. Plasma Process. Polym. 15:e1800121 10.1002/ppap.201800121

[B32] Mansurnezhad R. Ghasemi-Mobarakeh L. Coclite A. M. Beigi M. H. Gharibi H. Werzer O.. (2020). Fabrication, characterization and cytocompatibility assessment of gelatin nanofibers coated with a polymer thin film by initiated chemical vapor deposition. Mater. Sci. Eng. C 110:110623. 10.1016/j.msec.2019.110623 32204065

[B33] Mao X. Liu A. Tian W. Wang X. Gleason K. K. Hatton T. A. (2018). Enhancing Performance stability of electrochemically active polymers by vapor-deposited organic networks. Adv. Funct. Mater. 28:1706028 10.1002/adfm.201706028

[B34] Mao Y. Gleason K. K. (2004). Hot filament chemical vapor deposition of poly(glycidyl methacrylate) thin films using tert-butyl peroxide as an initiator. Langmuir 20, 2484–2488. 10.1021/la0359427 15835714

[B35] Martin T. P. Sedransk K. L. Chan K. Baxamusa S. H. Gleason K. K. (2007). Solventless surface photoinitiated polymerization: grafting chemical vapor deposition (gCVD). Macromolecules 40, 4586–4591. 10.1021/ma070150v

[B36] Matsumura H. Umemoto H. Gleason K. K. Schropp R. E. I. (2019). Catalytic Chemical Vapor Deposition. Weinheim: Wiley. 10.1002/9783527818655

[B37] McInnes S. J. P. Szili E. J. Al-Bataineh S. A. Vasani R. B. Xu J. Alf M. E.. (2016). Fabrication and characterization of a porous silicon drug delivery system with an initiated chemical vapor deposition temperature-responsive coating. Langmuir 32, 301–308. 10.1021/acs.langmuir.5b03794 26654169

[B38] McInnes S. J. P. Szili E. J. Al-Bataineh S. A. Xu J. Alf M. E. Gleason K. K.. (2012). Combination of iCVD and porous silicon for the development of a controlled drug delivery system. ACS Appl. Mater. Interfaces 4, 3566–3574. 10.1021/am300621k 22720638

[B39] Moni P. Al-Obeidi A. Gleason K. K. (2017). Vapor deposition routes to conformal polymer thin films. Beilstein J. Nanotechnol. 8, 723–735. 10.3762/bjnano.8.76 28487816 PMC5389201

[B40] Montero L. Baxamusa S. H. Borros S. Gleason K. K. (2009). Thin hydrogel films with nanoconfined surface reactivity by photoinitiated chemical vapor deposition. Chem. Mater. 21, 399–403. 10.1021/cm802737m18783272

[B41] Muralter F. Coclite A. M. Werzer O. (2019). Wrinkling of an enteric coating induced by vapor-deposited stimuli-responsive hydrogel thin films. J. Phys. Chem. C 123, 24165–24171. 10.1021/acs.jpcc.9b07340 31602284 PMC6778969

[B42] Muralter F. Perrotta A. Coclite A. M. (2018). Thickness-dependent swelling behavior of vapor-deposited smart polymer thin films. Macromolecules 51, 9692–9699. 10.1021/acs.macromol.8b02120 30591733 PMC6300310

[B43] Oh M. S. Song Y. S. Kim C. Kim J. You J. B. Kim T. S.. (2016). Control of reversible self-bending behavior in responsive Janus microstrips. ACS Appl. Mater. Interfaces 8, 8782–8788. 10.1021/acsami.5b12704 26974225

[B44] O'Schaughnessy W. S. Murthy S. K. Edell D. J. Gleason K. K. (2007). Stable biopassive insulation synthesized by initiated chemical vapor deposition of poly(1,3,5-trivinyltrimethylcyclotrisiloxane). Biomacromolecules 8, 2564–2570. 10.1021/bm070242s 17591748

[B45] Ozaydin Ince G. Demirel G. Gleason K. K. Demirel M. C. (2010). Highly swellable free-standing hydrogel nanotube forests. Soft Matter 6, 1635–1639. 10.1039/c000569j

[B46] Ozaydin-Ince G. Gleason K. K. Demirel M. C. (2011). A stimuli-responsive coaxial nanofilm for burst release. Soft Matter 7, 638–643. 10.1039/C0SM00922A

[B47] Park S. W. Lee D. Lee H. R. Moon H. J. Lee B. R. Ko W. K.. (2015). Generation of functionalized polymer nanolayer on implant surface via initiated chemical vapor deposition (iCVD). J. Colloid Interface Sci. 439, 34–41. 10.1016/j.jcis.2014.10.018 25463173

[B48] Peppas N. A. Hilt J. Z. Khademhosseini A. Langer R. (2006). Hydrogels in biology and medicine: from molecular principles to bionanotechnology. Adv. Mater. 18, 1345–1360. 10.1002/adma.200501612

[B49] Perrotta A. Werzer O. Coclite A. M. (2018). Strategies for drug encapsulation and controlled delivery based on vapor-phase deposited thin films. Adv. Eng. Mater. 20:1700639 10.1002/adem.201700639

[B50] Pryce Lewis H. G. Bansal N. P. White A. J. Handy E. S. (2009). HWCVD of polymers: commercialization and scale-up. Thin Solid Films 517, 3551–3554. 10.1016/j.tsf.2009.01.114

[B51] Randall G. C. Gonzalez L. Petzoldt R. Elsner F. (2018). An evaporative initiated chemical vapor deposition coater for nanoglue bonding. Adv. Eng. Mater. 20:1700839 10.1002/adem.201700839

[B52] Sayin S. Ozdemir E. Acar E. Ince G. O. (2019a). Multifunctional one-dimensional polymeric nanostructures for drug delivery and biosensor applications. Nanotechnology 30:412001. 10.1088/1361-6528/ab2e2c 31347513

[B53] Sayin S. Tufani A. Emanet M. Genchi G. G. Sen O. Shemshad S.. (2019b). Electrospun Nanofibers with pH-responsive coatings for control of release kinetics. Front. Bioeng. Biotechnol. 7:309. 10.3389/fbioe.2019.00309 31828065 PMC6892405

[B54] Schroder S. Polonskyi O. Strunskus T. Faupel F. (2020). Nanoscale gradient copolymer films via single-step deposition from the vapor phase. Mater. Today 37, 35–42. 10.1016/j.mattod.2020.02.004

[B55] Sevgili E. Karaman M. (2019). Initiated chemical vapor deposition of poly(hydroxypropyl methacrylate) thin films. Thin Solid Films 687:137446 10.1016/j.tsf.2019.137446

[B56] Shi X. Ye Y. Wang H. Liu F. Wang Z. (2018). Designing pH-responsive biodegradable polymer coatings for controlled drug release via vapor-based route. ACS Appl. Mater. Interfaces 10, 38449–38458. 10.1021/acsami.8b14016 30360069

[B57] Suh H. S. Kim D. H. Moni P. Xiong S. Ocola L. E. Zaluzec N. J.. (2017). Sub-10-nm patterning via directed self-assembly of block copolymer films with a vapour-phase deposited topcoat. Nat. Nanotechnol. 12, 575–581. 10.1038/nnano.2017.34 28346456

[B58] Tenhaeff W. E. Gleason K. K. (2009). Surface-tethered pH-responsive hydrogel thin films as size-selective layers on nanoporous asymmetric membranes. Chem. Mater. 21, 4323–4331. 10.1021/cm9023474

[B59] Tufani A. Ince G. O. (2015). Permeability of small molecules through vapor deposited polymer membranes. J. Appl. Polym. Sci. 132:42453 10.1002/app.42453

[B60] Unger K. Resel R. Coclite A. M. (2016). Dynamic studies on the response to humidity of poly (2-hydroxyethyl methacrylate) hydrogels produced by initiated chemical vapor deposition. Macromol. Chem. Phys. 217, 2372–2379. 10.1002/macp.201600271

[B61] Unger K. Salzmann P. Masciullo C. Cecchini M. Koller G. Coclite A. M. (2017). Novel light-responsive biocompatible hydrogels produced by initiated chemical vapor deposition. ACS Appl. Mater. Interfaces 9, 17408–17416. 10.1021/acsami.7b01527 28475310

[B62] Wang M. Wang X. Moni P. Liu A. Kim D. H. Jo W. J.. (2017a). CVD polymers for devices and device fabrication. Adv. Mater. 29:1604606. 10.1002/adma.201604606 28032923 PMC7161753

[B63] Wang M. Zhao J. Wang X. Liu A. Gleason K. K. (2017b). Recent progress on submicron gas-selective polymeric membranes. J. Mater. Chem. A 5, 8860–118886. 10.1039/C7TA01862B

[B64] Werzer O. Tumphart S. Keimel R. Christian P. Coclite A. M. (2019). Drug release from thin films encapsulated by a temperature-responsive hydrogel. Soft Matter 15, 1853–131859. 10.1039/C8SM02529K 30698598 PMC6390694

[B65] Yagüe J. L. Gleason K. K. (2012). Systematic control of mesh size in hydrogels by initiated chemical vapor deposition. Soft Matter 8, 2890–2894. 10.1039/c2sm07137a

[B66] Yang R. Asatekin A. Gleason K. K. (2012). Design of conformal, substrate-independent surface modification for controlled protein adsorption by chemical vapor deposition (CVD). Soft Matter 8:31 10.1039/C1SM06334K

[B67] Yang R. Gleason K. K. (2012). Ultrathin antifouling coatings with stable surface zwitterionic functionality by initiated chemical vapor deposition (iCVD). Langmuir 28, 12266–12274. 10.1021/la302059s 22873558

[B68] Yang R. Xu J. Ozaydin-Ince G. Wong S. Y. Gleason K. K. (2011). Surface-tethered zwitterionic ultrathin antifouling coatings on reverse osmosis membranes by initiated chemical vapor deposition. Chem. Mater. 23, 1263–1272. 10.1021/cm1031392

[B69] Yilmaz K. Sakalak H. Gürsoy M. Karaman M. (2019). Initiated chemical vapor deposition of poly(ethylhexyl acrylate) films in a large-scale batch reactor. Ind. Eng. Chem. Res. 58, 14795–14801. 10.1021/acs.iecr.9b02213

[B70] Yin J. Yagüe J. L. Eggenspieler D. Gleason K. K. Boyce M. C. (2012). Deterministic order in surface micro-topologies through sequential wrinkling. Adv. Mater. 24, 5441–5446. 10.1002/adma.201201937 22915065

[B71] Yu S. B. Baek J. Choi M. Oh Y. Lee H. R. Yu S. J.. (2016). Polymer thin films with tunable acetylcholine-like functionality enable long-term culture of primary hippocampal neurons. ACS Nano 10, 9909–9918. 10.1021/acsnano.6b03527 27792310

[B72] Yu S. J. Pak K. Kwak M. J. Joo M. Kim B. J. Oh M. S. (2018). Initiated chemical vapor deposition: a versatile tool for various device applications. Adv. Eng. Mater. 20:1700622 10.1002/adem.201700622

[B73] Zhao J. Gleason K. K. (2020). Solvent-less vapor-phase fabrication of membranes for sustainable separation processes. Engineering 6, 1432–1442. 10.1016/j.eng.2020.05.002

